# Enzymatic Cascades for Tailored ^13^C_6_ and ^15^N Enriched Human Milk Oligosaccharides

**DOI:** 10.3390/molecules24193482

**Published:** 2019-09-25

**Authors:** Thomas Fischöder, Samanta Cajic, Valerian Grote, Raphael Heinzler, Udo Reichl, Matthias Franzreb, Erdmann Rapp, Lothar Elling

**Affiliations:** 1 Laboratory for Biomaterials, Institute for Biotechnology and Helmholtz-Institute for Biomedical Engineering, RWTH Aachen University, Pauwelsstraße 20, 52074 Aachen, Germany; 2 Max Planck Institute for Dynamics of Complex Technical Systems, Sandtorstraße 1, 39106 Magdeburg, Germany; 3 Institute of Functional Interfaces, Karlsruhe Institute of Technology, 76131 Karlsruhe, Germany; 4 Chair of Bioprocess Engineering, Otto-von-Guericke-University, Universitätsplatz 2, 39106 Magdeburg, Germany; 5 glyXera GmbH, Leipziger Straße 44, 39120 Magdeburg, Germany

**Keywords:** glycobiology, biocatalysis, human milk oligosaccharides, isotopic labeling, quantification: nucleotide sugars, lacto-*N*-biose type, lacto-*N*-neo type, glycosyltransferases, capillary gel electrophoresis, MALDI-MS

## Abstract

Several health benefits, associated with human milk oligosaccharides (HMOS), have been revealed in the last decades. Further progress, however, requires not only the establishment of a simple “routine” method for absolute quantification of complex HMOS mixtures but also the development of novel synthesis strategies to improve access to tailored HMOS. Here, we introduce a combination of salvage-like nucleotide sugar-producing enzyme cascades with *Leloir*-glycosyltransferases in a sequential pattern for the convenient tailoring of stable isotope-labeled HMOS. We demonstrate the assembly of [^13^C_6_]galactose into lacto-*N*- and lacto-*N*-neo-type HMOS structures up to octaoses. Further, we present the enzymatic production of UDP-[^15^N]GlcNAc and its application for the enzymatic synthesis of [^13^C_6_/^15^N]lacto-*N*-neo-tetraose for the first time. An exemplary application was selected—analysis of tetraose in complex biological mixtures—to show the potential of tailored stable isotope reference standards for the mass spectrometry-based quantification, using matrix-assisted laser desorption/ionization-time of flight mass spectrometry (MALDI-TOF-MS) as a fast and straightforward method for absolute quantification of HMOS. Together with the newly available well-defined tailored isotopic HMOS, this can make a crucial contribution to prospective research aiming for a more profound understanding of HMOS structure-function relations.

## 1. Introduction

It has become increasingly apparent that human milk oligosaccharides (HMOS) consist of a cocktail of complex glycans and play important biological roles well beyond simple nutrition. Most of HMOS are involved in very complex and important biological mechanisms [1,2], protecting the gastrointestinal tract against toxin and viral and bacterial adhesion, mediating immune modulations and stimulating the brain development and several more [1,2,3]. HMOS are present in large quantities and are the third most abundant group of compounds in human milk, directly after lactose and lipids [3,4,5]. They display a unique and extremely high structural diversity [2,3,6,7], with approximately 150 elucidated structures so far [8].

Interestingly, it has been found that biological functions depend on both, the specific structure and the quantity of the HMOS present [8,9]. Striking is also their remarkable variability in composition and concentration of milk oligosaccharides (milk OS) during lactation depending on the donor phenotype [10]. Therefore, methods for absolute quantification of milk OS are of high interest and a prerequisite for the deeper understanding of the biological roles of HMOS. Moreover, a rising amount of HMOS is authorized as a nutrition supplement, which promotes an urgent future request for a rapid and robust “routine” quantification method for suitable quality control [11,12,13].

However, absolute quantification of milk OS has been proven to be a challenging task. The most commonly applied chromatographic, electrophoretic, and spectrometric methods often deliver only relative quantification of milk OS [14,15,16]. One reason for limited attempts to quantify diverse and complex milk OS is a small number of available standards. Matrix-assisted laser desorption/ionization-time of flight mass spectrometry (MALDI-TOF-MS) methods combined with stable isotope labeling have already been successfully applied for absolute quantification of other complex OS like *N*-glycans [17]. Nevertheless, this technique also relies on the availability of isotope-labeled internal standards [17,18,19]. Improved access to tailored isotopically labeled milk OS would enable the application of fast and straightforward MALDI-TOF-MS-based methods for absolute quantification of milk OS, suitable also for a complex sample matrix-like human milk.

Beside chemical routes for the HMOS synthesis [20,21], the enzyme-mediated HMOS tailoring is a very straightforward strategy [7,21,22,23,24,25]. *Leloir*-glycosyltransferases are especially known for their quantitative substrate conversion, a very high specificity, and high regioselectivity by utilizing activated nucleotide sugars [26]. Availability of recombinant enzymatic tools like a β1,4-galactosyltransferase-1 (β4GalT) [27,28], a β1,3-*N*-acetylglucosaminyltransferase (β3GlcNAcT) [29,30,31], and a β1,3-galactosyltransferase (β3GalT) [32,33] nowadays enables an efficient enzymatic synthesis approach towards linear HMOS. Elongation of lactose with *N*-acetyllactosamine (Gal(β1-4)GlcNAc-, LacNAc type 2) or lacto-*N*-biose (Gal(β1-3)GlcNAc-, LacNAc type 1) represents a basic motif of linear and branched HMOS. More precisely, the LacNAc units have to be β1-3-linked for the correct biomimetic structure of linear HMOS while additional β1-6-linked LacNAc units form the backbone of branched (*iso*-)HMOS [1].

Nevertheless, the *Leloir*-glycosyltransferases-mediated, isotopically labeled HMOS synthesis depends on the availability of the isotope-labeled nucleotide sugars as uridine-5´-diphospho-α-d-galactose (UDP-Gal) or uridine-5´-diphospho-α-d-*N*-acetylglucosamine (UDP-GlcNAc). Synthesis approaches of isotope-labeled nucleotide sugars are not mentioned very frequently in literature. An in vitro synthesis of ^14^C-enriched UDP-[^14^C]Gal was demonstrated already 50 years ago using an uridyltransferase [34]. Virgilio et al. incorporated ^13^C-labeled galactose by the enzymatic synthesis in *poly-*LacNAc type II tetra- and hexaoses. The synthesis principle was also demonstrated for the synthesis of non-labeled linear lacto-*N*-neo-type HMOS and therefore postulated as a possible way towards isotopically labeled HMOS [35]. A chemoenzymatic strategy for the synthesis of UDP-[^2^H]GlcNAc starting from the sugar-1-phosphate was performed using the uridylyltransferases activity of the bifunctional *Escherichia coli* (*E.coli*) enzyme GlmU [36]. Recent developments of cascades with salvage pathway enzymes and their use in novel synthesis strategies can provide the nucleotide sugars UDP-Gal and UDP-GlcNAc in a much more efficient way [37,38,39]. Furthermore, recent studies impressively demonstrate the value of defined isotopically labeled OS for in vivo research applications [40].

Here, we utilize a previously described strategy in a very efficient synthesis set-up for the production of UDP-[^15^N]GlcNAc and UDP-[^13^C_6_]Gal on a 200 and a 400 µmol scales. [39]. As previously demonstrated, nucleotide sugars are readily accessible for *Leloir-*glycosyltransferases-mediated glycan synthesis without further purification steps [32]. In this way, we demonstrate the synthesis of linear ^13^C_6_-labeled Lacto-*N*-type and Lacto-*N*-neo-type HMOS up to octaoses as well as ^13^C_6_ and ^15^N-labeled lacto-*N*-neo-tetraose ([^13^C_6_/^15^N]LNnT), to the best of our knowledge, for the first time in a sequential enzymatic synthesis cascade. Production in a 125 µmol scale of [^13^C_6_/^15^N]LNnT facilitates its application for absolute MALDI-TOF-MS-based quantification of tetraose in milk samples as a proof-of-concept exercise to show the potential utility of tailored stable isotope-labeled HMOS for quantitative studies.

## 2. Results and Discussion

### 2.1. Nucleotide Sugar Synthesis

Synthesis of milk OS requires building blocks—monosaccharides. In order to be used in oligosaccharide synthesis mediated by *Leloir-*glycosyltransferases, these monosaccharides need to be activated first to a high-energy donor form—nucleotide sugars. Production of isotope-labeled nucleotide sugars was carried out as described before, employing cascades of salvage pathway enzymes in a repetitive batch approach (Supplementary Materials, Scheme S1) [39].

**Scheme 1:**
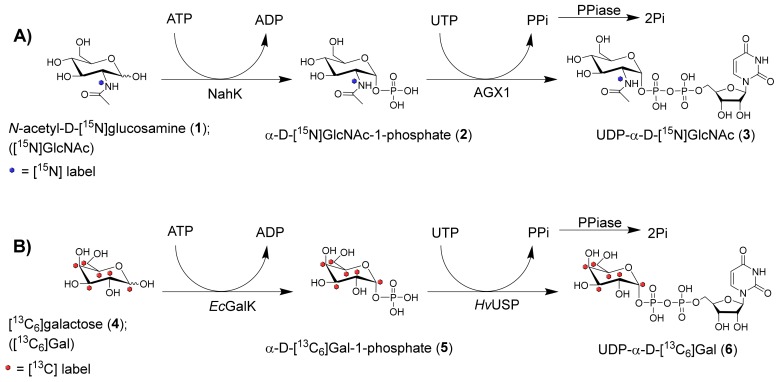
Repetitive batch synthesis for (**A**) isotope-labeled UDP-[^15^N]GlcNAc (**3**) by the *N*-acetylhexosamine-1-kinase (NahK), UDP-GlcNAc pyrophosphorylase (AGX1), and an inorganic pyrophosphatase (PPiase) and (**B**) isotope-enriched UDP-[^13^C_6_]Gal (**6**) by galactokinase (*Ec*GalK), the UDP-sugar pyrophosphorylase from *Hordeum vulgare* (*Hv*USP) and PPiase.

Briefly, within the nucleotide sugar-producing cascades, the monosaccharides (**1** or **4**) were first phosphorylated by the respective ATP-dependent kinases (*N*-acetylhexosamine-1-kinase from *Bifidobacterium longum* (NahK) or *E.coli* galactokinase (*Ec*GalK)). The corresponding UDP-sugar pyrophosphorylases (UDP-GlcNAc pyrophosphorylase (AGX1) or UDP-sugar pyrophosphorylase from *Hordeum vulgare* (*Hv*USP)) catalyse the UTP-dependent UDP-sugar synthesis from the respective sugar-1-phosphates (**2** or **5**), and an inorganic pyrophosphatase (PPiase) hydrolyses the inhibiting by-product pyrophosphate (PP_i_) (Scheme 1). Regarding the repetitive batch synthesis strategy, a centrifugal concentrator was utilized to carry out the enzymatic synthesis and for the subsequent product separation of the respective batch. Each new cycle of the repetitive batch approach started with the addition of fresh substrate to the enzymes, remaining in the concentrator (Supplementary Materials, Scheme S1). From a commercial point of view, isotope-labeled monosaccharide substrates are rather precious. With this in mind, the synthesis conditions for compounds **3** and **6** were carefully adjusted to increase the product yield. By pre-tempering of all reaction solutions and elongation of the reaction time to one hour, we were able to obtain full substrate conversion (Table 1). Product concentrations were determined by capillary electrophoresis with UV detection (CE-UV) (data not shown) and the product verification was carried out via electrospray ionization mass spectrometry (ESI-MS) as previously reported (Table 1; Supplementary Materials, Figures MS1, and MS2) [32,37,39]. The non-labeled nucleotide sugars utilized in this study were available from our earlier work [39] (Supplementary Materials, Figures MS3, and MS4). The synthesized compound **6** (UDP-[^13^C_6_]Gal) contains isotopically enriched galactose with a stable ^13^C incorporated at each carbon position of the hexose which causes the mentioned mass difference of 6 compared to the natural compound (UDP-Gal). Accordingly, incorporation of the stable isotope ^15^N in GlcNAc causes a one-unit mass difference in compound **3** when compared to the non-labeled UDP-GlcNAc.

The direct salvage enzyme cascades utilized in this study are characterized in previous works by great space-time yields and high stability under optimized reaction conditions [23,38,39]. Additionally, their application into a repetitive batch approach maximizes the enzyme productivities and reduces, therefore, enzyme preparation time and effort to the minimum (Supplementary Materials, Table S1). As demonstrated in previous studies for the synthesis of a set of non-labeled nucleotide sugars, this synthesis approach is highly reproducible and can be easily scaled up to a multi-gram scale without extensive laboratory effort [39]. Due to a complete conversion, unreacted sugars and intermediates were not present, while nucleotides can be straightforwardly degraded, using an alkaline phosphate. As previously demonstrated, obtained nucleotide sugar solutions are ready to use for a subsequent *Leloir-*glycosyltransferases-mediated glycan synthesis [32].

### 2.2. HMOS Synthesis

**Scheme 2:**
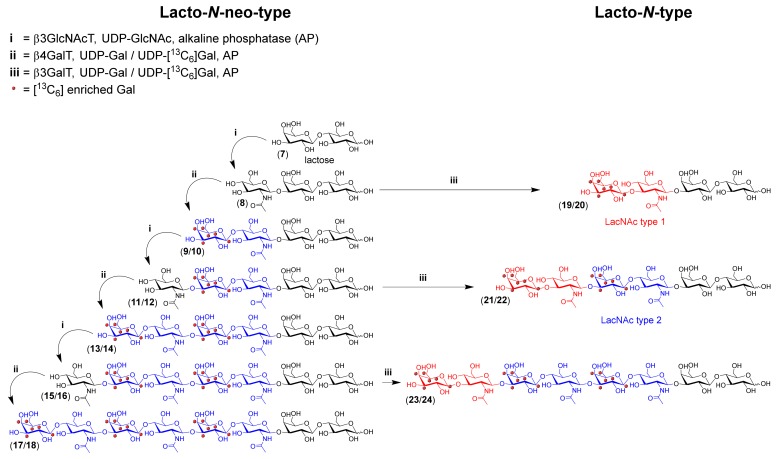
Sequential enzymatic synthesis of human milk oligosaccharides (HOMS). Compounds **10**, **12**, **14**, **16**, **18**, **20**, **22**, and **24** contain ^13^C_6_-enriched galactoses in each LacNAc unit. Compounds **8**, **9**, **11**, **13**, **15**, **17**, **19**, **21,** and **23** were synthesized as unlabeled HMOS.

The synthesized nucleotide sugars were used without any further purification step in a sequential synthesis of ^13^C_6_-labeled and non-labeled linear HMOS with type I or type II LacNAc extensions of the lactose core as depicted in Scheme 2. The respective reaction progress was monitored by capillary electrophoresis with laser-induced fluorescence detection (CE-LIF). Suitable internal standards for migration time alignment allowed for product assignment including the challenging linkage isomers (Supplementary Materials, Figures S1–S5).

**Figure 1:**
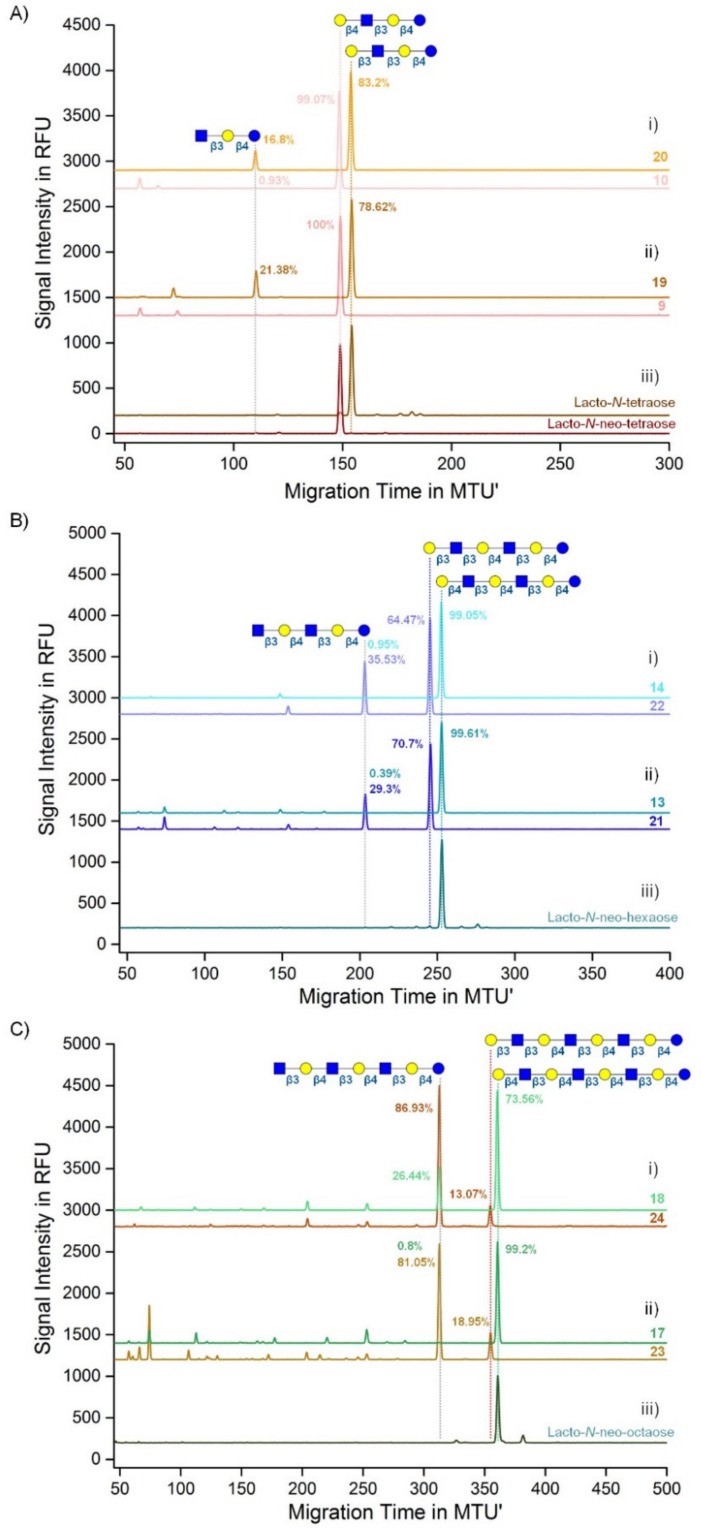
Multiplexed capillary gel electrophoresis with laser-induced fluorescence detection (xCGE-LIF) analysis of the linear HMOS synthesis products: tetraoses (**A**), hexaoses (**B**), and octaoses (**C**). Upper electropherograms show (**i**) ^13^C_6_ galactose-enriched compounds **10, 14**, **18**, **20**, **22,** and **24** and (**ii**) centered native compounds **9**, **13**, **17**, **19**, **21**, and **23**. The bottom electropherograms display (**iii**) the migration time matching with commercially available standards. Lacto-*N*-neo-type HMOS were analyzed after 24 h reaction time, while the final reaction step for Lacto-*N*-type (terminal β3Gal) compounds was carried out after 72 h. Symbolic representation of oligosaccharide structures is drawn with GlycoWorkbench v.1.0.3353 by the guidelines of the Consortium for Functional Glycomics: blue circle, glucose; blue square, *N*-acetylglucosamine; yellow circle, galactose [48,49].

Product characterization and determination of relative conversion rates were carried out using more sensitive, high-performance multiplexed capillary gel electrophoresis with laser-induced fluorescence detection (xCGE-LIF). xCGE-LIF has proven as an ideal separation tool for oligosaccharide analysis, enabling rapid separation and detection of isomeric compounds, even with small differences in the monosaccharide composition, positions, and linkages [16,41,42,43,44,45,46]. This technique can be employed in a multi-capillary format (up to 96 capillaries), making the method high-throughput and thereby appropriate for enzyme reaction monitoring and product identification [47]. The normalized migration times of synthesized isotopically labeled HMOS were compared to the normalized migration times of commercial native HMOS standards if available (Figure 1). Identification and annotation of OS were carried out primarily based on our internal HMOS database (comparison of migration times). Identification and annotation of synthesized HMOS by comparison of migration times with in-house database entries (consisting of commercially available standards and previously annotated structures) was feasible for all synthesized structures since a wide range of linear HMOS, even the ones with minor structural differences, were extensively characterized and annotated previously [23]. Compositions of products were verified by LC-ESI-MS and MALDI-TOF-MS (Supplementary Materials, Figures MS5–MS17 and MTS1–MTS7).

Formation of the synthesis intermediates (GlcNAc-terminated structures) was pushed to a quantitative conversion respectively by the addition of fresh enzyme and elongation of the reaction time up to 72 h if necessary (Supplementary Materials, Figure S1). With respect to the known specificity, the production of Lacto-*N*-triose II (GlcNAc-β1,3-Gal-β1,4Glc) has to be considered critically. The β3GlcNAcT is known for good conversion of longer *poly*-LacNAc type II motives [30,32] but also for a bad acceptance of structures without *N*-acetyl group [23,50]. Nevertheless, we were able to reach 92.3% conversion of lactose within 48 h and nearly full conversion (99.7%) after 68 h reaction time as exemplarily depicted in Figure S6 (Supplementary Materials). The *N*-acetyllactosamine type II core (Galβ1-4GlcNAc) was synthesized by the β3GlcNAcT from *Helicobacter pylori* and the human β4GalT which are already known for a good substrate conversion in *poly*-LacNAc syntheses [30].

Synthesis of Lacto-*N*-type HMOS (Galβ1-3GlcNAc) was carried out by the *E.coli* β3GalT (WbgO) as described previously [32]. The acceptor’s structure differs from the known sugar structures occurring in the *E.coli* O55:H7 *O*-antigen repeating unit [33]. Here, the substrate acceptance could be one important reason why we were not able to push the enzymatic reaction to a quantitative conversion (Figure 1B,C). We assumed further an enzymatic limitation, especially for longer structures, which was also observed in the previously published sequential synthesis of *poly-*LacNAc type I [32]. Regarding the lacto-*N*-neo-type HMOS synthesis, we mainly achieved good conversions (Figure 1; Supplementary Materials, Figures MTS1–MTS5). Surprisingly, we obtained only a yield of 73% within the last sequential synthesis step for compound **18** ([^13^C_6_]lacto-*N*-neo-octaose). On the contrary, we obtained nearly full conversion (99.5%) for the synthesis of compound **17** with nearly the same reaction conditions (Figure 1; Supplementary Materials, Figure MTS6). Therefore, we assumed no enzymatic or structural limitation in this synthesis step especially since no substrate length limitation for the used human β4GalT is known from the previous studies [30,32,51]. Therefore, we rated the enzymatic elongation toward longer HMOS as feasible, possibly after a clean-up which removes reaction residues from earlier steps. Since the scope of this study focuses on a semi-sequential synthesis set-up without purification steps and we were not aiming for even longer structures, we did not investigate this further.

**Scheme 3:**
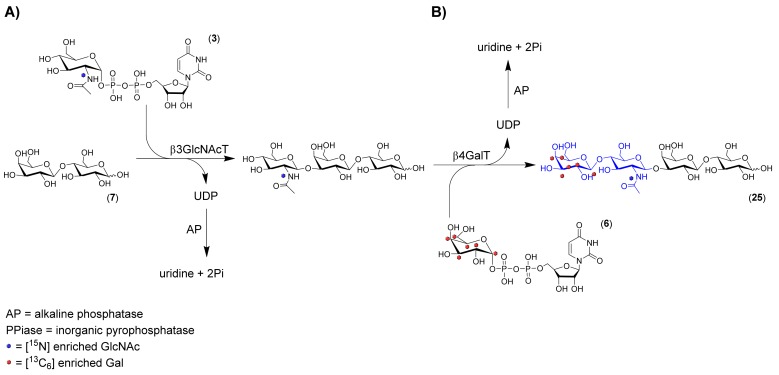
Sequential enzymatic synthesis cascade for ^13^C_6_ and ^15^N-labeled Lacto-*N*-neo-type tetraose (compound **25**). (**A**) Synthesis of Lacto-*N*-triose II using previously synthesized UDP-[^15^N]GlcNAc as a substrate. (**B**) Synthesis of [^13^C_6/_^15^N]LNnT, [^13^C_6_]Gal(β1,4)[^15^N]GlcNAc(β1,3)Gal(β1,4)Glc using previously synthesized UDP-[^13^C_6_]Gal.

The sequential synthesis of ^13^C_6_ and ^15^N-labeled lacto-*N*-neo-tetraose ([^13^C_6_/^15^N]LNnT) depicted in Scheme 3 delivers an overall synthetic yield (lactose-based) of 91.6% (Supplementary Materials, Figures S6 and MTS7 (purified compound **25**)). Starting from the isotopically labeled monosaccharides [^13^C_6_]Gal and [^15^N]GlcNAc, the incorporation yield was calculated to be 74.2% overall synthetic steps. Hereby a slight excess of nucleotide sugars used for the transferases-mediated synthesis steps to [^13^C_6_/^15^N]LNnT affects the incorporation yield. Nevertheless, we rated the introduced synthesis strategy as a very efficient approach. In addition, we achieved the synthesis of [^13^C_6_/^15^N]LNnT in a multi-µmol scale (106 µmol). For its application as an internal standard in the quantification of milk OS, it was necessary to confirm that the compound was obtained in good quality, with no other milk OS in the sample that could compromise the purity and consequently quantification accuracy. Purity and identity of the isotopically enriched standard were confirmed by xCGE-LIF and MALDI-TOF-MS (Supplementary Materials, Figure MTS7). We observed the desired compound only and no lactose or triose after purification by an ultra-dialysis step. Additionally, to determine the number of OS, it was necessary that the standard used is quantitative. Therefore, an aliquot of the synthesized and purified [^13^C_6_/^15^N]LNnT isotope-standard solution was estimated by total organic carbon (TOC) analysis (Supplementary Materials, Figure TOCS1, and Table TOCS1). Data obtained enabled us to apply the compound as a quantitative standard for a proof-of-concept exercise showing the potential utility of enzymatically synthesized standards for quantification of HMOS, even in a complex matrix like milk.

### 2.3. Absolute Compound Quantification in Complex Samples by MS

There is an increasing amount of reports showing that biological functions of HMOS depend not only on the specific structure of an HMOS but also on the quantity of the HMOS [52,53,54,55,56]. Therefore, in order to better understand all the effects of HMOS on infant health and risk of diseases, it is important to be able to quantify these compounds. Accurate, sensitive, fast, and straightforward methods that can quantify these compounds even in complex matrixes ranging from body fluids to food formulas will be essential for future studies.

Besides chromatography (e.g., high-performance liquid chromatography with UV or fluorescence detection and high-pH anion-exchange chromatography with pulsed amperometric detection) [57,58,59,60,61,62,63,64,65,66] and electrophoresis (e.g., CE-UV and CGE-LIF) [16,67,68,69,70], MS has emerged as one of the methods of choice for the elucidation and characterization of milk OS from various sources because of its high resolving power, specificity, and selectivity, which is the result of differentiation of compound based on specific masses and from the fragmentation specific to linkage and monosaccharide sequence [15,71,72,73,74]. MALDI-MS has become a fast, simple, and convenient method for the analysis of even complex HMOS mixtures [75,76,77]. However, studies on the analysis of HMOS using MALDI-MS have been mainly qualitative as the absolute quantification is compromised by structure-dependent ionization efficiencies, ion suppression in complex samples, fluctuations in instrument performance, and variability in matrix crystallization. These sources of variability in MALDI-TOF-MS can be greatly diminished by the use of stable isotope-labeled standards. Briefly, due to the introduced mass difference, there is no overlap in the spectra between native and stable isotopes-labeled analytes. Both the analyte and its isotopic pair have equal ionization efficiency so that, when spiked to the analyte-containing sample in a known concentration, the isotope-labeled internal standard acts as a molecular balance. Given that the molar ratio of the isotopologues is within the linear range of the so-called response factor, one can easily compare their signal intensity or area under the curve (AUC) and deduce absolute quantities. Absolute quantification by MS employing stable isotope-labeled internal standards has been a routine method in metabolomics and proteomics and is recently introduced for quantification of another group of complex OS—*N*-glycans [17,78,79,80,81,82]. In contrast, quantification of milk OS by MS has been hampered largely by a lack of isotope-labeled HMOS standards. This study aimed to overcome this issue by an enzymatic synthesis of isotope-labeled HMOS.

**Figure 2:**
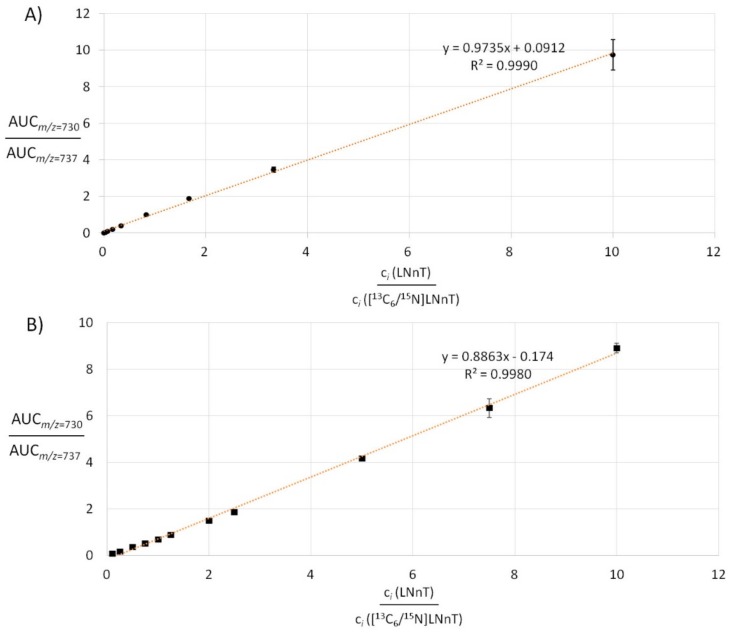
Calibration plots for the response function of LNnT to [^13^C_6_/^15^N]LNnT by matrix-assisted laser desorption/ionization-time of flight mass spectrometry (MALDI-TOF-MS). [^13^C_6_/^15^N]LNnT was mixed commercial LNnT in H_2_O (**A**) and bovine H-milk (**B**), covering a broad spectrum of concentrations (0.25 µM to 1.5 mM). The graph displays averaged replicates with error bars representing the standard deviation (*n* > 3). LNnT and [^13^C_6_/^15^N]LNnT were purified from bovine H-milk (Weinstephan, 3.5% fat) by porous graphitized carbon-solid-phase extraction (PGC-SPE).

As one example for the use of these enzymatically synthesized heavy isotope-labeled HMOS standards, we applied [^13^C_6_/^15^N]LNnT for the MALDI-TOF-MS-based absolute quantification of commercial LNnT spiked into homogenized bovine milk. In comparison to human milk, bovine milk contains qualitatively and quantitatively fewer OS (25 identified of the 39 reported) [83,84]. Nevertheless, it was essential to prove that there was no detectable tetraose in commercial bovine milk used as a matrix to be spiked with commercial and synthesized isotope-labeled LNnT. (Supplementary Materials, Figure MTS8). The 7 Da mass increment of [^13^C_6_/^15^N]LNnT over the native compound LNnT facilitated a clear separation of the isotopic profiles, which was a necessary prerequisite for an accurate quantification (Supplementary Materials, Figure MTS9). To evaluate the response factor and dynamic range of the MALDI-TOF-MS analysis, LNnT and [^13^C_6_/^15^N]LNnT were analyzed with varying molarities (Figure 2A). The coefficient of determination (R^2^) of 0.999 confirms the linear response for relative molarities of LNnT to [^13^C_6_/^15^N]LNnT from 0.1 to 10. Furthermore, the response factor and dynamic range were investigated taking into account the complete sample preparation workflow (Figure 2B). The linear regression (R^2^ = 0.998) for samples extracted from bovine milk is in great accordance with the direct analysis of LNnT and [^13^C_6_/^15^N]LNnT. The data indicates no negative effect of the sample preparation for absolute quantification.

**Figure 3:**
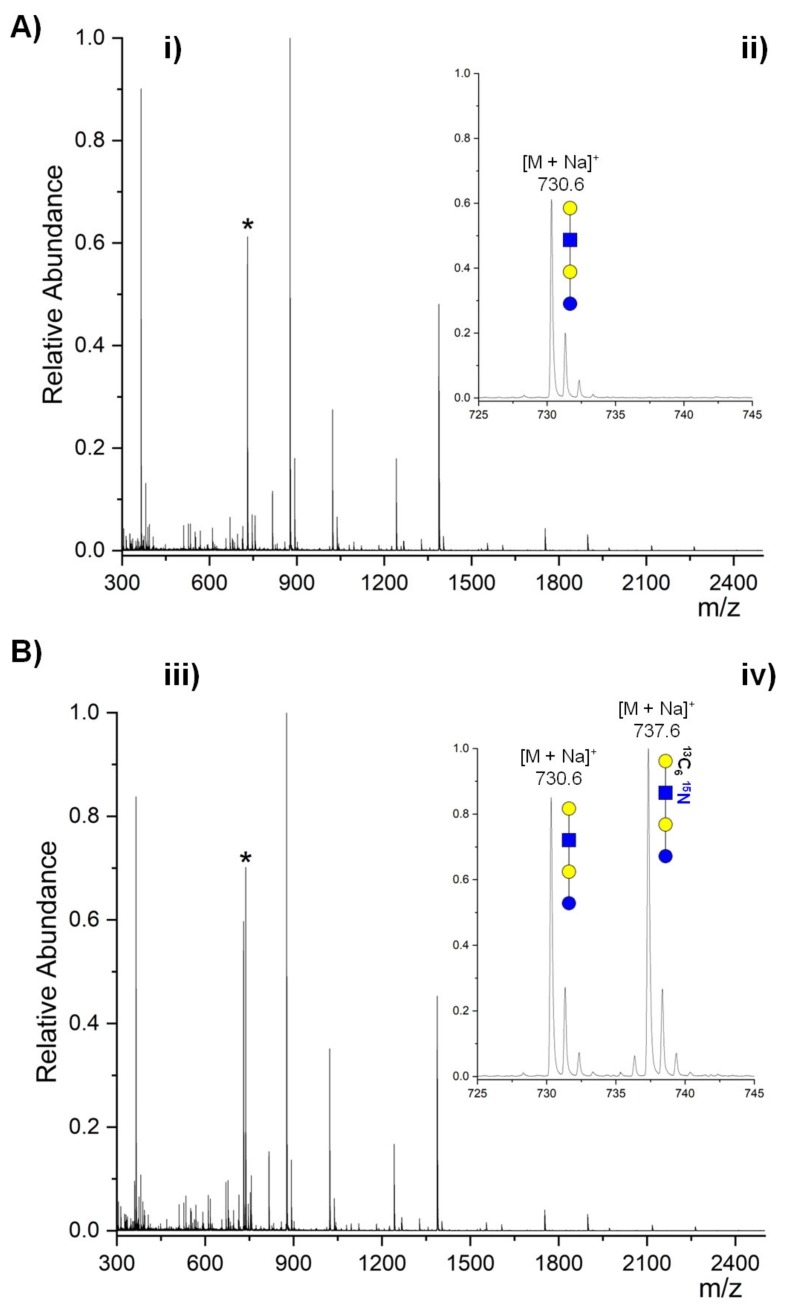
(**A**) MALDI-TOF mass spectrum of PGC-SPE extracted human milk in the *m*/*z* range from 300 to 2500 (**i**) and the magnified *m*/*z* range from 726 to 746 (**ii**). An asterisk indicates the signal corresponding to tetraose (*m*/*z* = 730.6204 [M + Na]^+^). (**B**) MALDI-TOF mass spectrum from PGC-SPE extracted human milk spiked with [^13^C_6_/^15^N]LNnT. An asterisk in the in the *m*/*z* range from 300 to 2500 (**iii**) indicates the signals corresponding to tetraose (*m*/*z* = 730.6204 [M + Na]^+^ and [^13^C_6_/^15^N] LNnT (737.641 [M + Na]^+^). The two isotopologues are highlighted in the amplified m/z range from 726 to 746 (**iv**).

In order to challenge the method in a more complex matrix, we used [^13^C_6_/^15^N]LNnT as an internal standard for the quantification of tetraose in a human milk sample, collected 153 days postpartum. As complexity and oligosaccharide content of human milk far exceeds the one from bovine milk, with some reporting at least 1000 different OS in human milk [77], it was necessary to examine that there were no compounds interfering with the quantification. Therefore, OS extracted from the human milk sample were analyzed without the addition of the isotope-labeled standard (Figure 3A). The zoom into the *m*/*z* range from 726 to 746 (Figure 3A**-ii**) shows not only the expected isotopic distribution of tetraose (monoisotopic peak indicated by an asterisk) [85] but also an otherwise flat baseline. These findings approve the eligibility of [^13^C_6_/^15^N]LNnT as an internal standard for MALDI-TOF-MS-based quantification of tetraose in human milk. In an analogous manner to the aforementioned results, different quantities of human milk (0.25 to 3 µL) were diluted in H_2_O and spiked with 1 µL [^13^C_6_/^15^N]LNnT (1 mM). A representative measurement is depicted in Figure 3B**-iii** and Figure 3B**-iv**, showing human milk-derived tetraose (*m*/*z* of 730.6 [M + Na]^+^) and the enzymatically synthesized standard (*m*/*z* of 737.6 [M + Na]^+^) with a mass difference of 7 Da (based on singly charged species). The early incorporation of the internal standard into the human milk sample resulted in improved reproducibility and robustness of the quantification, due to the identical treatment of target compound and standard. This advantageous feature was confirmed by the linear response function (R^2^ = 0.9927) of tetraose and [^13^C_6_/^15^N]LNnT, extracted from human milk (Supplementary Materials, Figure S7). Based on linear regression, a tetraose concentration of 1.33 mM was determined for the human milk sample (153 days postpartum). The exemplarily shown utilization of 1 µL internal standard (1 mM) enables the precise quantification of tetraose from human milk over a relative concentration range of more than 1 order of magnitude (application of 0.25 to 3 µL human milk). Therefore, the enzymatically tailored HMOS evade the need for time- and labor-intensive preliminary experiments to find adequate standard concentrations for different sample sets.

Nevertheless, the applied MALDI-TOF-MS-based method has some drawbacks, like the inability to distinguish the linkage isomers lacto-*N*-tetraose (LNT) and LNnT, both present in human milk [2,6]. While there are techniques like the analysis of post-source decay fragment ions and instruments such as ion-mobility MS, which are facilitating additional resolution, these approaches demand not only appropriate equipment but also certain expertise. However, the use of isotope-labeled standards while incorporating a high-resolution separation step prior to MS should increase information gained and improves the quantification process by separating various isomers that cannot be differentiated by mass alone.

Results obtained by different groups analyzing concentrations of LNnT and LNT reported in human milk vary considerably. Such variation may be attributed to the general biological variability as well as to other biological parameters like gestational age, Lewis blood group, secretor status of the mother, lactation period, or analytical methods [8,86]. All this variability makes it difficult to compare the presented results with other studies. LNT and LNnT are naturally occurring tetrasaccharides belonging to the group of non-fucosylated neutral HMOS. It was shown that they could protect against important systemic infections of the newborn [9,87,88,89]. In addition, they act as natural prebiotics, promoting the growth of beneficial gut microbiota (bifidobacteria and lactobacilli), thereby suppressing the growth of undesirable bacteria [90,91,92,93]. LNnT is, besides 2´-fucosyllactose (2′-FL), the only synthetically produced oligosaccharide approved by the European Food Safety Authority (EFSA) for supplementation to infant formula [12]. For these reasons, a fast, accurate, and sensitive method that can quantify these OS will be very useful for future studies.

Another reported approach which is taking advantage of the mass difference between the light and heavy form of an oligosaccharide is based on the incorporation of stable isotopes into OS by reductive amination [18,94,95]. One sample is reduced with sodium borohydride, and another is decreased with sodium borodeuteride, and afterward, two samples are mixed in a 1:1 (*v*/*v*) ratio. The ratios of light and heavy isotope-labeled OS are then used to compare the relative intensities between the two samples. Limitation of this approach is that only the relative quantification is achieved and, due to overlapping isotopic signals of unlabeled and labeled OS, analysis becomes more difficult. With the availability of isotopically labeled HMOS standards, the MALDI-TOF-MS-based approach can position itself as a fast, simple, sensitive, and precise tool for HMOS quantification, even in a complex sample.

## 3. Materials and Methods

### 3.1. Recombinant Enzyme Preparation

Expression and subsequent purification of the recombinant transferases: the human β1,4-galactosyltransferase-1 (β4GalT), the *E.coli* O55:H7 β1,3-galactosyltransferase (β3GalT), and a the β1,3-N-acetylglucosaminyltransferases from *Heliobacter pylori* (β3GlcNAcT) were carried out as described previously [23,27,29,30,32]. For the β3GalT and the β3GlcNAcT, a buffer exchange was carried out by dialysis (10 kDa cut-off). The β3GalT dialysis buffer contained 100 mM NaH_2_PO_4_ at pH 7.5, 500 mM NaCl, and 5 mM DTT. The β3GlcNAcT dialysis buffer consisted of 20 mM Tris-HCl at pH 7.4, 200 mM NaCl, 1 mM EDTA and 1 mM DTT. The purified enzymes were stored at 4 °C. Heterologous kinases and pyrophosphorylases expression, as well as purification, were done as published before [37,38,39]. *Ec*GalK, *Hv*USP, NahK, and the human AGX1 were subsequently diafiltrated and concentrated in a 30 kDa centrifugal concentrator (Vivaspin® 20) from Sartorius (Göttingen). Therefore, a 50 mM Tris/HCl buffer (pH 7.5) was used. *Ec*GalK and AGX1 were stored at −20 °C, and β4GalT, β3GalT, β3GlcNAcT, NahK, and *Hv*USP were stored at 4°C after the buffer exchange.

### 3.2. Enzyme Activity Assay

Kinases and pyrophosphorylases activity tests were carried out in a 96-well plate format on a multiplexed capillary electrophoresis system with UV detection (MP-CE (UV)) as described before [37,38,39]. Briefly, the reactions were carried out in a volume of 300 µL and at 37 °C. The reaction mixture for NahK contained 1 U pyrophosphatase from *Saccharomyces cerevisiae* (Sigma-Aldrich (Roche)), 2 mM GlcNAc, 2 mM ATP, and 2 mM MgCl_2_ and was buffered by 50 mM Tris(HCl) at pH 8.0. The AGX1 reaction mixture was also buffered by a Tris(HCl) system (pH 8.0) and supplemented with 2 mM GlcNAc-1-P, 2 mM MgCl_2_, 2 mM UTP, and 1 U pyrophosphatase. For the *Ec*GalK reaction mixture, 2 mM Gal, 2 mM MgCl_2_, 2 mM ATP, and 1 U pyrophosphatase were buffered at pH 7.5 by 50 mM Tris(HCl), while the *Hv*USP mixture contained 1 U pyrophosphatase, 2mM Gal-1-P, 2 mM MgCl_2_, and 2 mM UTP in a 50 mM Tris(HCl) buffer system (pH 7.5). The reactions were stopped at different time points by the addition of a termination solution, containing sodium dodecyl sulfate (SDS), to a final concentration of 7 mM to denature the enzymes immediately. Internal normalization standards, 4-aminobenzoic acid (PABA) and 4-aminophthalic acid (PAPA), were added to the sample solution together with the termination solution to a final concentration of 1 mM, respectively. The peak areas were normalized to PABA, and the concentrations were determined via calibration curves as formerly published [37,38,39]. Kinases activities were determined by increasing ADP concentrations. While the pyrophosphorylases activities were determined by the UDP-sugar increase over time. The quantification of these UV-active compounds was carried out as formerly described by Wahl et al. at a wavelength of 254 nm [37]. More precisely, a calibration curve was recorded for the peak area of commercial standards normalized to the peak area of 1 mM *para*-amino benzoic acid (PABA) plotted against the varying standard concentration. Analytes concentrations were deduced from the linear slope area, calculated with the obtained normalized peak area. Therefore, one µmol substrate conversion per minute was defined as 1 U (enzyme unit).

Transferases activity assays were carried out and analyzed on CE-LIF as described previously [23]. Shortly, all reaction mixtures were incubated at 30 °C and contained 6.5 mM MnCl_2_, 6.5 mM MgCl_2_ as well as 6 U alkaline phosphatase (AP) (ThermoFischer) buffered by 100 mM Tris(HCl) at pH 7.5. Each 5 mM of the respective acceptor substrate, 6.5 mM of the respective nucleotide sugar, and 10 µg purified enzyme were supplemented to start the reactions. More precisely, lactose and UDP-GlcNAc were used for the β3GlcNAcT assay, and Lacto-*N*-triaose and UDP-Gal were used for the β4GalT and β3GalT assays. The enzyme reactions were stopped by heating up small samples (6 µL, 95 °C for 5 min) on different time points. After centrifugation, the supernatant (2 µL) was used for fluorescent labeling (8-aminopyrene-1,3,6-trisulfonic acid (APTS)), post-derivatization hydrophilic interaction chromatography-solid phase extraction (HILIC-SPE) sample clean-up and analysis via CE-LIF as described in the Method section and previous studies [23,43]. The volumetric activity (U/mL) was calculated from the linear slope area.

### 3.3. HMOS Analysis via CE-LIF and xCGE-LIF

CE-LIF was mainly used to determine the transferases activity, as described above, and follow the HMOS synthesis reaction. APTS labeling, HILIC-SPE clean-up, and CE-LIF measurement were carried out as described before [23,43]. Additionally, commercial maltose, cellooctaoses, and maltononaoses were spiked as internal migration time normalization standards. High-resolution and -sensitivity xCGE-LIF analytics were used for a structure verification based on a migration time matching to the in-house database and commercially available standards [39]. Sample preparation including APTS labeling, HILIC-SPE, and further xCGE-LIF measurement and analysis was carried out as detailed described before [42,43].

### 3.4. Nucleotide Sugar Synthesis

UDP-[^13^C_6_]galalactose and UDP-*N*-acetyl-[^15^N]glucosamine were produced as published before in a repetitive batch mode using a centrifugal concentrator device (Vivaspin®) as a reactor and separation device [39]. For the synthesis, commercially available isotopically labeled monosaccharides (Sigma/Campo and Omicron Biochemicals) were used. To receive an optimal conversion, all reaction solutions were pre-tempered, and the synthesis reaction time was elongated to each one h in total. The concentration was determined as described before by MP-CE (UV) analysis [37,38,39]. Briefly, the product concentrations were determined by the peak area normalized to the internal standard PABA and calculated using a calibration curve of the corresponding commercial non-isotope-labeled nucleotide sugars.

### 3.5. Sequential ^13^C_6_ and Native HMOS Synthesis

For the sequential synthesis of compound **8**, the initial reaction mixture was buffered by 100 mM Tris/HCl (pH 7.5). The mixture contains further 25 mM KCl, 1 mM MgCl_2_, 1 mM MnCl_2_, 1 mM lactose (compound **7**), 1 mM in-house produced UDP-GlcNAc, and 20 mU/mL AP. β3GlcNAcT (50 mU/mL) was added to start the reaction in a total volume of 2 mL, respectively. The reaction was incubated at 30 °C for at least 24 h. The product formation was monitored by CE-LIF as described in the corresponding section. If necessary, the reaction time was elongated to 72 h and fresh enzyme and nucleotide sugar were supplemented after 24 h. Removal of the enzymes between the sequential steps was carried out by an ultra-filtration step using a centrifugal concentrator as recommended by the supplier (Vivaspin®500 from Sartorius, 10 kDa). Ongoing sequential product elongation with constant buffer and compound compositions (mentioned above) was started by the addition of AP, the respective transferase (250 mU/mL β4GalT, 150 mU/mL, β3GalT mU/mL, or 50 mU/mL β3GlcNAcT) and the respective in-house produced nucleotide sugar. Defined lengths of HMOS (target compounds **10**, **14**, **18**, **20**, **22, 24** and **9**, **13**, **17**, **19**, **21, 23**) were synthesized by alternating addition and removal of the transferases β3GlcNAcT, β4GalT, or β3GalT and the respective in-house synthesized nucleotide sugar starting from lactose. The progress of each enzymatic elongation step was monitored by CE-LIF and additional structural characterization was performed by xCGE-LIF, LC-ESI-MS, and MALDI-TOF-MS, as described elsewhere in this paper.

### 3.6. Sequential Synthesis of ^13^C_6_ and ^15^N-Labeled Lacto-N-Neo-Tetraose

Synthesis of ^13^C_6_ and ^15^N-labeled LNnT (compound **25**, [^13^C_6_]Gal-β1,4-[^15^N]GlcNAc- β-1,3-Gal-β-1,4-Glc) was carried out on a 125 µmol scale. The reaction contained 6.25 mM MgCl_2,_ 25 mM KCl, 5.5 mM pre-synthesized UDP-[^15^N]GlcNAc, 5 mM lactose, and 1 mM DTT in a Tris/HCl buffer system (pH 7.5). Addition of 100 mU/mL β3GlcNAcT and 20 mU/mL AP started the reaction. After 48 h at 30 °C, the intermediate formation was monitored by CE-LIF as described in the corresponding section, which took additional 20 h (labeling, clean-up, and measurement). Afterward, the reaction was stopped by heat inactivation of the involved enzymes for 5 minutes at 95 °C. Further elongation was carried out by the addition of 6.25 mM MgCl_2_, 250 mU/mL β4GalT, 20 mU/mL AP, and 5.5 mM pre-synthesized UDP-[^13^C_6_]Gal. The reaction was stopped by a heat-inactivation step (5 min/95 °C) after an additional 48 h incubation at 30°C and the final product formation was analyzed by CE-LIF. The [^13^C_6_/^15^N]LNnT was pre-treated for further experiments by ultra-dialysis. Briefly, enzymes were removed after heat precipitation by centrifugation. Low-molecular-synthesis compounds were removed by at least 3 times low-cut-off (100–500 Da, FloatALyser® G2) dialysis against ultrapure water overnight monitored by a conductivity meter. The samples were dried in a vacuum concentrator at 60 °C overnight and resolved in ultrapure MilliQ water.

### 3.7. TOC Analysis of the Purified [^13^C_6_/^15^N]Lacto-N-Neo-Tetraose

The organic carbon content of the samples was determined by a Sievers M9 TOC Analyzer (GE Analytical Instruments, Manchester, United Kingdom) by measuring the difference between the total carbon (TC) and the total inorganic carbon (TIC). TC of the sample was wet-chemical oxidized with UV radiation forming CO_2_ which passed a CO_2_ permeable membrane for detection by conductometry. TIC was reacted to CO_2_ using phosphoric acid and passed the same CO_2_-permeable membrane for detection by conductometry. The TOC value was given by the difference between these two values: TOC = TC – TIC. The measurement was conducted at a sample flow of 0.5 mL/min while adding 1 µL/min phosphoric acid (6 M) and 2.8 µL/min ammonium persulfate solution (15% *w*/*w*) for a working range up to 50 ppm TIC and 10 ppm TOC. The detection range was specified by the manufacturer from 0.03 ppb to 50 ppm with a precision of ±2%. The initial calibration was done with potassium hydrogen phthalate (Merck KGaA, Darmstadt, Germany, 1.09017.0100) and was linear in a range from carbon-free samples up to 10 ppm organic carbon. Of the samples, 1 mg was weighed with a Mettler AT 261 Delta range balance (±0.02 mg accuracy) and dissolved in 10 mL ultrapure water. These solutions were diluted 10 and 20 times with ultrapure water and measured against the same ultrapure water as blank. For validation of the method, the carbon content of α-Lactose Monohydrate (Merck KGaA, Darmstadt, Germany) was calculated by measuring the corresponding TOC and dividing it by the initial sample weight. The agreement between the experimental value (39.67% w/w) and the theoretical value (39.97% *w*/*w*) shows the high accuracy of the method.

### 3.8. MS-Based Analysis

All experiments, including sample preparation steps for MS measurements, were carried out using LC-MS-grade water and chemicals if not stated otherwise. Qualitative characterization of the synthesized HMOS standards was carried out as described previously [23]. All enzymatically synthesized HMOS samples were prepared for MS analysis by ultra-dialysis as detailed described above for the [^13^C_6_/^15^N]LNnT pre-treatment.

### 3.9. Nucleotide Sugar and HMOS Analysis via ESI-MS

Mass verification of the nucleotide sugars in pre-treated samples was carried out as described previously [39]. Briefly, the remaining reaction residuals (nucleoside phosphates) were removed from the samples by an AP digest overnight at room temperature. Afterward, the enzymes were removed by an ultra-flirtation step using a centrifugal concentrator as recommended by the supplier (Vivaspin®500 from Sartorius, 10 kDa). Flow-through samples of prepared compounds **1**–**4** were partly supplemented with 0.1–0.5 µL ammonium hydroxide. Their subsequent analysis was performed on a Dionex HPLC system equipped with a Multospher 120 RP 18 HP-3µ HPLC column (60 mm × 2 mm) from CS-Chromatographie Service GmbH, Langerwehe. Therefore, an MS-grade ACN/water (50:50, *v*/*v*) mixture was used as a mobile phase with a flow rate of 0.2 mL min^−1^. Mass data were collected with a connected ESI-MS detector (Finnigan Surveyor MSQ Plus from Thermo Scientific) in a negative mode. The temperature was set to 400 °C, the needle voltage was set to −4 kV, and the cone voltage was set to 100 V.

The samples were supplemented with small amounts (0.1–0.5 µL) of ammonium hydroxide. Afterward, HMOS mass was analyzed using an LC-ESI-MS system. Therefore, a Dionex system was equipped with a polymer-based HILIC column (Shodex Asahipak NH2-50 4E (4.6 × 250 millimeter (mm))). As a mobile phase, an ACN:water gradient from 70% to 50% ACN over 15 minutes followed by an additional 5 minutes with an isocratic flow at 50% ACN (1 mL/min) was used. Mass data were collected using the conditions and the connected ESI-MS detector mentioned above for the nucleotide sugar analysis. All experiments for MS analysis were carried out with ultrapure water and LC-MS-grade ACN.

### 3.10. Extraction of OS from Human Milk

The human milk sample was collected 153 days postpartum using a manual milk pump and immediately stored at −20 °C. OS were isolated from human and bovine milk-based on a modified version of a previously reported workflow [75]. Therefore, 0.25 to 3 µL of human milk was diluted to 49 µL with H_2_O and spiked with 1 µL [^13^C_6_/^15^N]LNnT (1 mM). Bovine milk (10 µL 3.5% fat H-milk from Weihenstephan diluted to a final volume of 100 µL) was spiked with 1 µL [^13^C_6_/^15^N]LNnT (1 mM) and 0.1 to 10 µL commercial LNnT (1 mM) (Elicityl, Crolles, France). All samples were centrifuged at 14,000× *g* and at room temperature for 20 min using a Heraeus Fresco 17 microcentrifuge (Thermo Scientific, Bellefonte PA, USA). Subsequently, 60% of the aqueous phase was subjected to solid-phase extraction (SPE). For each sample, approximately 2 mg porous graphitic carbon (PGC) from prepacked 25 mg cartridges (Thermo Scientific, Bellefonte PA, USA) were loaded onto Pierce C18 100 µL tips (Thermo Scientific, Bellefonte PA, USA), placed in a 96-well PCR rack. To pass the liquid through the stationary phase, the rack was placed on a 96-deep well storage plate and centrifuged at 500× *g* and at room temperature for 1 min using a Heraeus Multifuge X1R (Thermo Scientific, Bellefonte PA, USA).

Equilibration of the stationary phase was achieved in two cycles of sequential application of 100 µL 40% (*v*/*v*) ACN_aq_ and 100 µL 20% (*v*/*v*) ACN_aq_ (both 0.1% (*v*/*v*) TFA_aq_), followed by 100 µL 0.1% (*v*/*v*) TFA_aq_ five times. After the application of approximately 60 µl sample, the PGC material was washed five times with 100 µL 0.1% (*v*/*v*) TFA_aq_. Following elution in 70 μL 40% (*v*/*v*) ACN_aq_ containing 0.1% (*v*/*v*) TFA_aq_ three times, all samples were dried for MALDI-TOF-MS analysis using a FRVC 2-33 CDplus rotational vacuum evaporator (Martin Christ, Osterode am Harz, Germany) and resolubilized in 10 µL H_2_O (extraction from human milk) or 20 µL H_2_O (extraction from bovine milk).

### 3.11. MALDI-TOF-MS Analysis and Absolute Quantification of HMOS

All MALDI-TOF mass spectra were acquired on an ultrafleXtreme MALDI-TOF/TOF-MS (Bruker Daltonics, Bremen, Germany) in reflectron positive ion mode. For each spectrum, 15,000 laser shots (50 shots per raster point in random walk) from a Smartbeam-II laser were averaged using a 120 ns delayed extraction time and a 25 kV accelerating voltage. For external calibration of the *m*/*z* range from 200 to 2500, a peptide standard mixture (Bruker Daltonics) and a dextran hydrolysate were used. Analyte ionization was facilitated by freshly prepared super-dihydroxybenzoic acid (S-DHB) (≥99.0%, Sigma-Aldrich, Steinheim, Germany) matrix (10 mg/mL) in 30% (*v*/*v*) ACN_aq_, 0.1% (*v*/*v*) TFA_aq_, and 4 mM NaCl. Therefore, 0.5 µL matrix solution was spotted onto an MTP AnchorChip 800/384 TF MALDI target (Bruker Daltonics) and immediately dried in a vacuum evaporator, followed by 0.5 µL sample spotted onto the matrix layer in an analogous manner.

All spectra were processed with flexAnalysis version 3.3 Build 80 (Bruker Daltonics), using the top-hat filter for baseline subtraction and a centroid algorithm for peak detection with 0.5 *m*/*z* peak width and a signal-to-noise ratio (SNR) threshold of 6. Processed spectra were exported in text format and transferred to OriginLab 2019 and Microsoft Excel for data evaluation. To determine the methods dynamic range and verify the direct proportionality of monoisotopic peak intensities from LNnT (*m*/*z* 730.6204 [M + Na]^+^) and [^13^C_6_/^15^N]LNnT (*m*/*z* 737.641 [M + Na]^+^), three calibration curves were prepared (one for each of the PGC-extracted samples (human and bovine milk) and one from a dilution series of synthesized [^13^C_6_/^15^N]LNnT with the commercial LNnT standard.

## 4. Conclusions

To further deepen our understanding of the functions of milk OS, it is essential to first increase our knowledge not only on the variety and complexity of these molecules in human milk but also on their quantities. The quantification of these structurally complex and diverse but still similar compounds is often limited by the small number of commercially available standards. Therefore, we strived to establish an approach to the enzymatic synthesis of stable isotope-labeled HMOS that can be employed as internal standards for quantitative analysis. We provided a straightforward synthesis strategy to isotopically labeled nucleotide sugars with high yields regarding the valuable monosaccharide substrates. Further, we introduced a flexible sequential synthesis approach to efficient tailoring of stable isotope [^13^C_6_]-labeled linear HMOS structures by the use of *Leloir*-glycosyltransferases. We demonstrated synthetic capabilities as well as the potential and limitations of the introduced strategy for selected HMOS up to octaoses. We further presented for the first time the enzymatic synthesis of ^15^N-enriched UDP-[^15^N]GlcNAc and its precise incorporation in a [^13^C_6_/^15^N]Lacto-*N*-neo-tetraose in a 106 µmol scale with an overall percentage yield (lactose-based) of 91.6%. Analysis of tetraose in complex milk mixtures was thought as a proof-of-concept exercise to show that MALDI-TOF-MS-based profiling method, when combined with well-defined ^13^C standards, can become a rapid, robust, and sensitive method for absolute quantification of milk OS.

In conclusion, the presented synthesis approach enables increased access to an even broader set of enzymatically tailored isotope-labeled HMOS than here described. Neutral HMOS (with type I and II extensions) represent basic structural motifs of many known HMOS which are accessible for further modifications, e.g., fucosylation or sialylation. Additionally, the sequential set-up combined with a purification step allows for precise incorporation of an isotopic sugar unit on a freely selectable position according to the respective demand. Tailored isotopically labeled HMOS could not only enable fast and straightforward quantitative MALDI-TOF-MS-based analysis, but even allow quantifying challenging linkage isomers of neutral HMOS when separation precedes detection by MS. The use of stable isotope-labeled HMOS standards may help to gain more information about the concentrations of OS in human milk or any other biofluid such as feces and urine, a crucial step towards a deeper understanding of HMOS structure-function relations and implementation to infant nutrition.

## Figures and Tables

**Table 1:** Analysis of the synthesized nucleotide sugars compounds **3** and **6** by capillary electrophoresis with UV detection (CE-UV) and electrospray ionization mass spectrometry (ESI-MS).

Compound	Amount [µmol]	Average Yield [%]	Calculated *m*/*z* [M − H]^−^	Observed *m*/*z* [M − H]^−^
**3** (UDP-[^15^N]GlcNAc)	200	100%	607.1 ^(a)^	607.2
**6** (UDP-[^13^C_6_]Gal)	400	100%	571.3 ^(a)^	570.9
UDP-GlcNAc ^(a)^			606.1	606.2
UDP-Gal ^(a)^			565.3 ^(a)^	564.9

**^(a)^** Provided from our previous study [39].

## Supplementary Materials

Additional CE-LIF electropherograms, as well as mass spectrograms, can be found in Supplementary Materials. Figures S1–S6 exemplarily depict the sequential synthesis progress and separation of challenging linkage isomers including the used internal migration time standards. Figures MS1–MS17 show ESI-MS mass spectrums of the synthesized products including nucleotide sugars and HMOS. Figures MTS1–MTS6 show MALDI-TOF-MS mass spectrums of the synthesized HMOS. Figure MTS7 shows xCGE-LIF analysis and MALDI-TOF-MS of the purified [^13^C_6/_^15^N]LNnT. Figure MTS7 shows the MALDI-TOF-MS mass spectrums of the complex sample matrix—bovine milk—before and after spiking of model analytes and the isotopic [^13^C_6/_^15^N]LNnT reference standard. Figure S7 shows the calibration plot for the response function to varying molar ratios of tetraose to [^13^C_6_/^15^N]LNnT in human milk.

## Abbreviations

2′-fucosyllactose (**2′-FL**); 4-aminobenzoic acid (**PABA**); 4-aminophthalic acid (**PAPA**); 8-aminopyrene-1,3,6-trisulfonic acid (**APTS**); alkaline phosphatase (**AP**); area under the curve (**AUC**); β1,3-galactosyltransferase (**β3GalT**); β1,3-*N*-acetylglucosaminyltransferase (**β3GlcNAcT**); β1,4-galactosyltransferase-1 (**β4GalT**); capillary electrophoresis with laser-induced fluorescence detection (**CE-LIF**); capillary electrophoresis with UV detection (**CE-UV**); electrospray ionization mass spectrometry (**ESI-MS**); European Food Safety Authority (**EFSA**); galactokinase (***Ec*GalK**); galactose (**Gal**); multiplexed capillary gel electrophoresis with laser-induced fluorescence detection (**xCGE-LIF**); lacto-*N*-tetraose (**LNT**); lacto-*N*-neo-tetraose (**LNnT**); liquid chromatography–electrospray ionization–mass spectrometry (**LC-ESI-MS**); human milk oligosaccharides (**HMOS**); matrix-assisted laser desorption/ionization mass spectrometry (**MALDI-MS**); matrix-assisted laser desorption/ionization time of flight mass spectrometry (**MALDI-TOF-MS**); oligosaccharides (**OS**); mass spectrometry (**MS**); multiplexed capillary electrophoresis system with UV detection (**MP-CE (UV)**); *N*-acetylglucosamine (**GlcNAc**); *N*-acetylhexosamine-1-kinase (**NahK**); *N*-acetyllactosamine (**LacNAc**); pyrophosphatase (**PPiase**); porous graphitized carbon-solid-phase extraction **(PGC-SPE**); pyrophosphate (**PPi**); signal-to-noise ratio (**SNR**); total carbon (**TC**); total inorganic carbon (**TIC**), total organic carbon (**TOC**); uridine-5′-diphospho-α-d-galactose (**UDP-Gal**); uridine-5′-diphospho-α-d-*N*-acetylglucosamine (**UDP-GlcNAc**); UDP-sugar pyrophosphorylase (**AGX1**); UDP-sugar pyrophosphorylase from *Hordeum vulgare* (***Hv*USP**).
