# Supplementary material for: Enzymatic Cascades for Tailored 13C6 and 15N Enriched Human Milk Oligosaccharides
Source: Molecules. 2019 Sep 25;24(19):3482. doi: 10.3390/molecules24193482 (PMC6803985; doi:10.3390/molecules24193482)
Supplement: Supplementary file 1 [file molecules-24-03482-s001.pdf]

molecules

# Supplementary Materials

# Enzymatic cascades for tailored $^{13}C_6$ and $^{15}N$ enriched human milk oligosaccharides

**Thomas Fischöder [1,†], Samanta Cajic [2,†], Valerian Grote [2], Raphael Heinzler [3], Udo Reichl [2,4], Matthias Franzreb [3], Erdmann Rapp [2,5,*] and Lothar Elling [1,*]**

[1] Laboratory for Biomaterials, Institute for Biotechnology and Helmholtz-Institute for Biomedical Engineering, RWTH Aachen University, Pauwelsstraße 20, 52074 Aachen, Germany; t.fischoeder@biotec.rwth-aachen.de

[2] Max Planck Institute for Dynamics of Complex Technical Systems, Sandtorstraße 1, 39106 Magdeburg, Germany; cajic@mpi-magdeburg.mpg.de, grote@mpi-magdeburg.mpg.de, udo.reichl@mpi-magdeburg.mpg.de

[3] Institute of Functional Interfaces, Karlsruhe Institute of Technology, Karlsruhe, Germany; raphael.heinzler@kit.edu, matthias.franzreb@kit.edu

[4] Otto-von-Guericke-University, Chair of Bioprocess Engineering, Universitätsplatz 2, 39106 Magdeburg, Germany

[5] glyXera GmbH, Leipziger Straße 44, 39120 Magdeburg, Germany

† These authors contributed equally to this work

* Correspondence: l.elling@biotec.rwth-aachen.de; Tel.: +49-241-80-28350; Fax: +49-241-80-22387

rapp@mpi-magdeburg.mpg.de; Tel.: +49-391-6110-314; Fax: +49-391-6110-535

## Nucleotide sugar synthesis

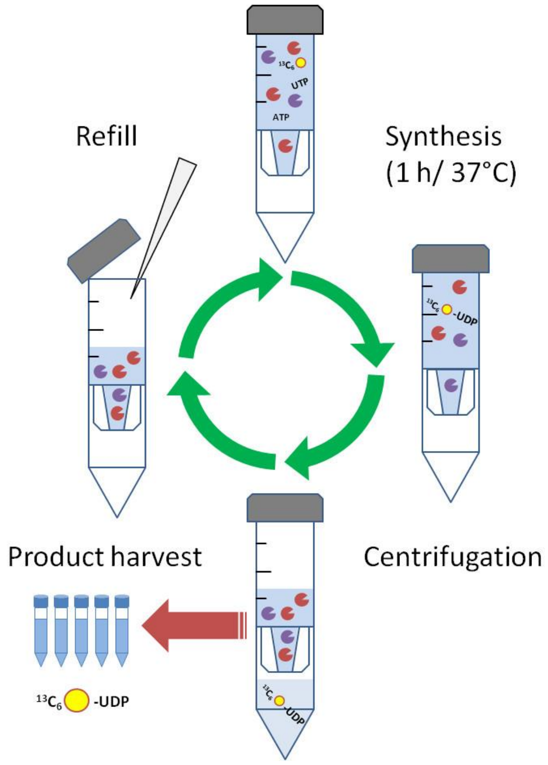


**Scheme S1.** Schematic depiction of the repetitive batch synthesis workflow.

**Table S1.** Calculated space-time-yield and mass base turnover number for the repetitive batch synthesis of compound **3** and **6**.

| compound | amount [µmol] | Space-time-yield [g/L*h] | Mass based turnover number [g $_{product}$/g $_{enzymes}$*]$^{-}$ |
|---|---|---|---|
| **3** (UDP-[$^{15}$N]GlcNAc) | 200 | 6.05 | 5.3 |
| **6** (UDP-[$^{13}C_6$]Gal) | 400 | 5.65 | 8.7 |

* consider all involved enzymes

**Capillary electrophoresis with laser laser-induced fluorescence detection (CE-LIF)**

Maltose and maltononaose were introduced as internal standards for a suitable migration time alignment, which allows a solid product assignment including the possibility to differentiate between the challenging linkage isomers (Figure S1-S3). Due to co-migration of lacto-*N*-neo-octaose with maltononaose, cellooctaose was used instead (Figure S4-S5).

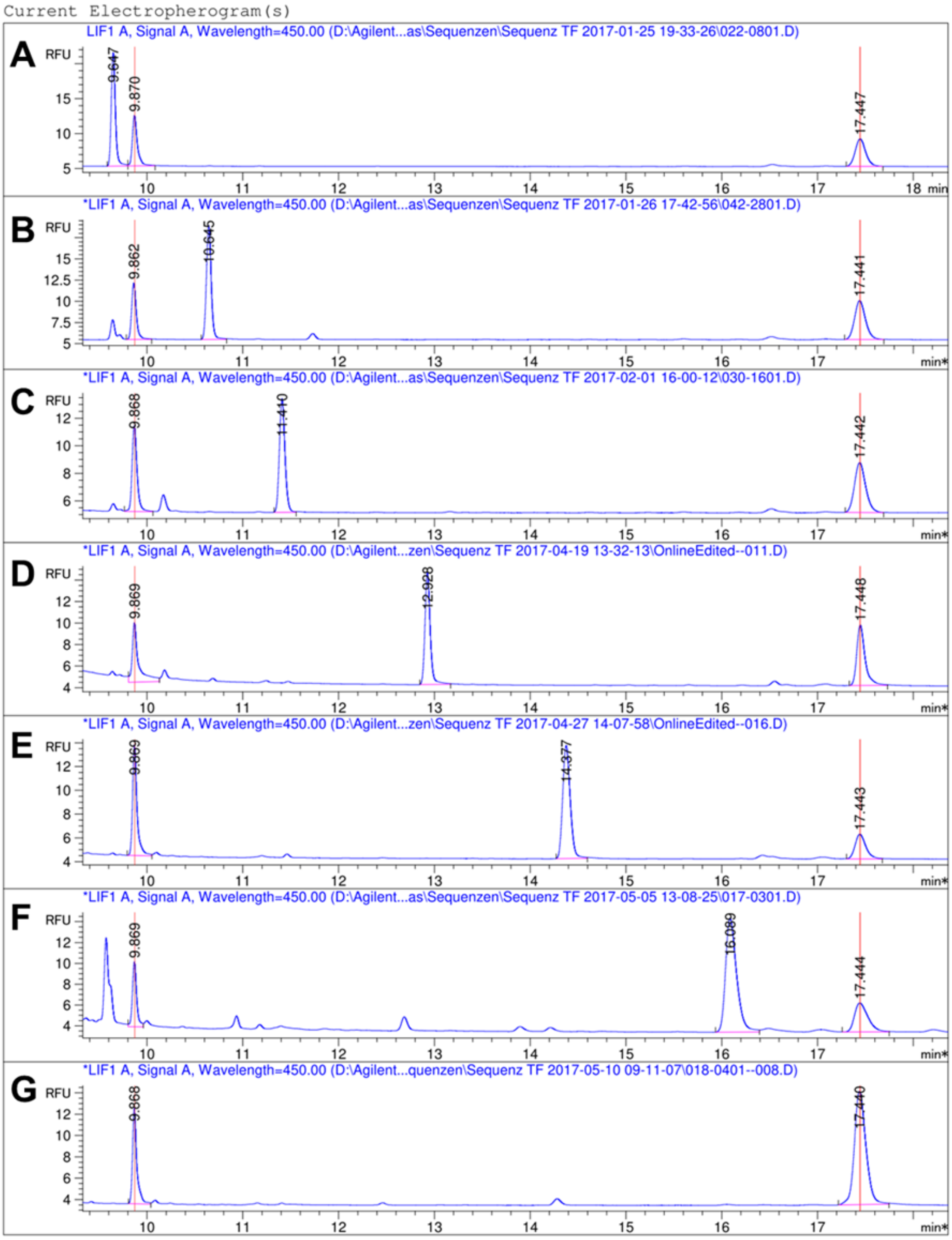


**Figure S1.** CE-LIF (normalized migration time) overlay of the lacto-*N*-neo-type HMOS synthesis, internal standard maltose (9.86), maltononaose (17.44). (**A**) Lactose, (**B**) lacto-*N*-neo- triaose, (**C**) tetraose, (**D**) pentaose, (**E**) hexaose (**F**) heptaose (**G**) octaose (without maltononaose).

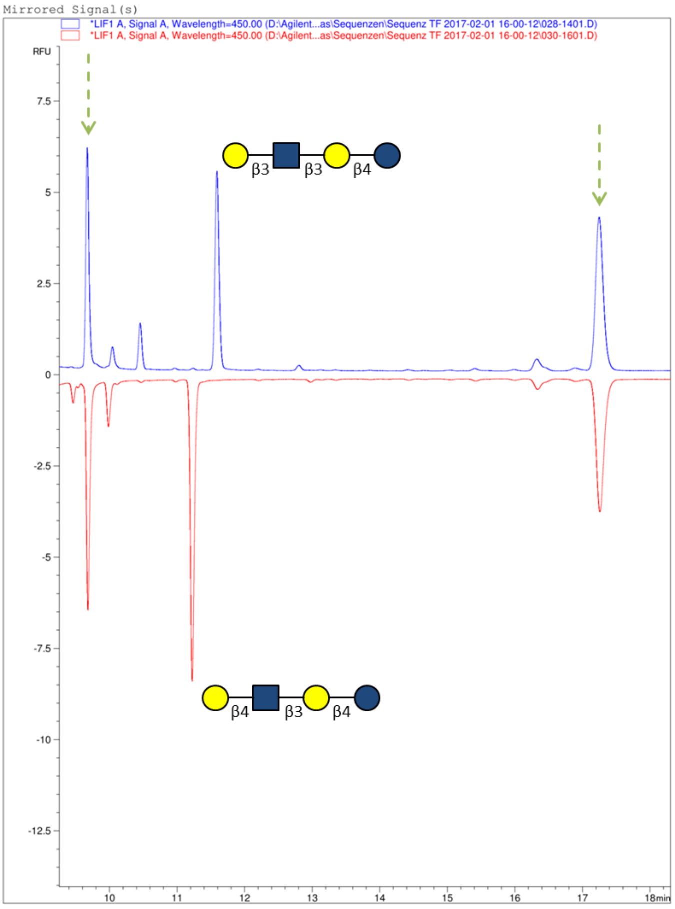


**Figure S2.** CE-LIF separation of lacto-*N*-neo-type and lacto-*N*-type tetraose (normalized migration time), internal normalization standards (green arrow) Maltose (9.86) and maltononaose (17.44). Synthesis of compound **10** (red, lacto-*N*-neo-tetraose, Gal(β1,4)GlcNAc(β1,3)Gal(β1,4)Glc) and compound **20** (blue, lacto-*N*-tetraose, Gal(β1,3)GlcNAc(β1,3)Gal(β1,4)Glc).

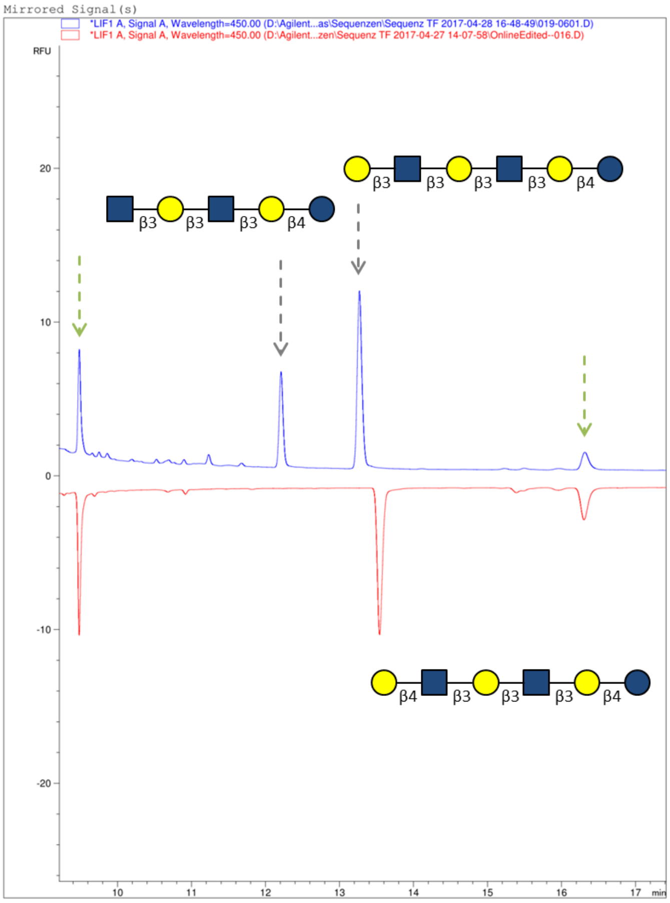


**Figure S3.** CE-LIF separation of lacto-*N*-neo-type and lacto-*N*-type hexaose (normalized migration time), internal normalization standards (green arrow) maltose (9.86) and maltononaose (16.6). Synthesis of compound **14** (red, lacto-*N*-neo-hexaose, Gal(β1,4)GlcNAc(β1,3)Gal(β1,4) GlcNAc(β1,3)Gal(β1,4)Glc) and compound **22** (blue, lacto-*N*-hexaose, Gal(β1,3)GlcNAc(β1,3) Gal(β1,4)GlcNAc(β1,3)Gal(β1,4)Glc).

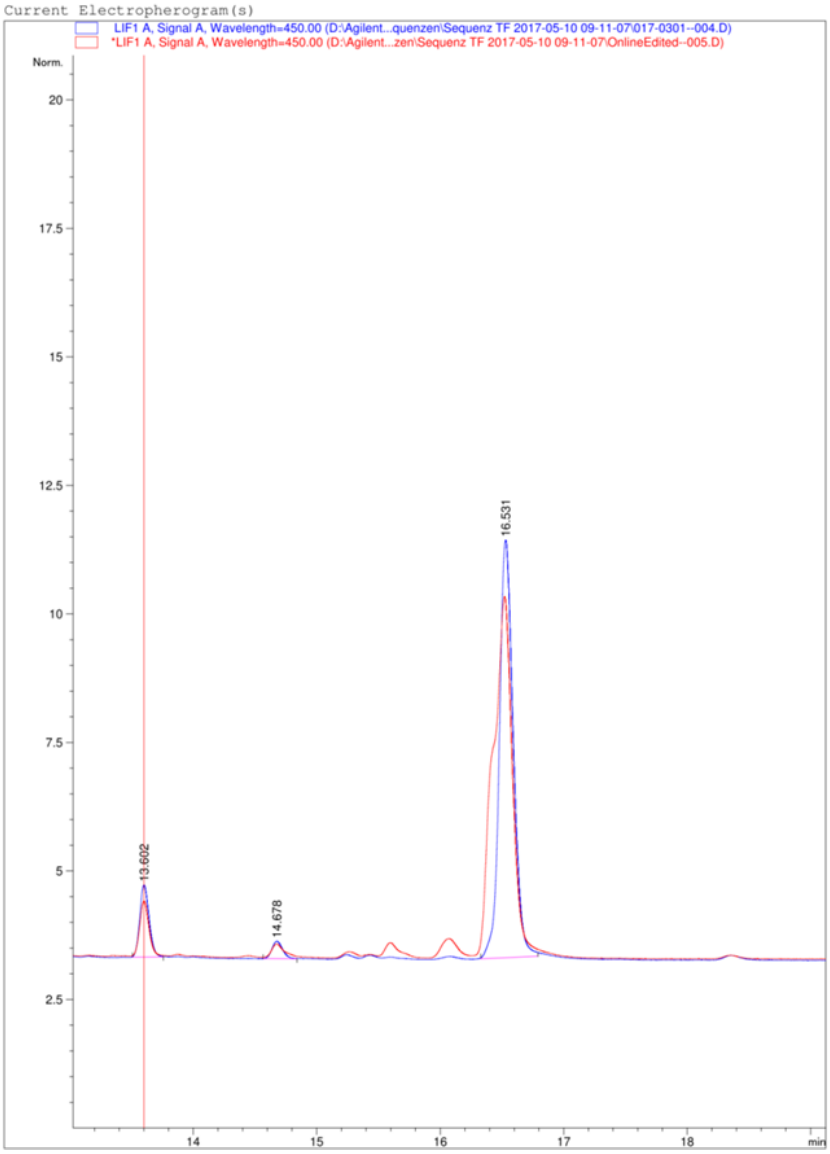


**Figure S4.** CE-LIF (normalized migration time), internal standard cellooctaose (13.60). Maltononaose (red, 16.53), lacto-*N*-neo-octaose (blue, 16.53).

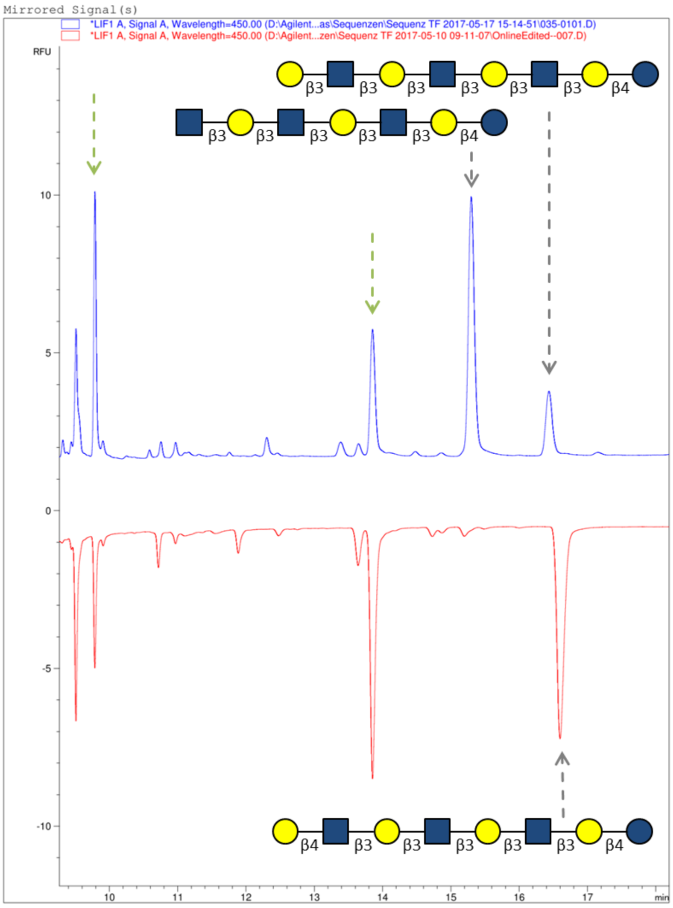


**Figure S5.** CE-LIF separation of lacto-*N*-neo-type and lacto-*N*-type octaose (normalized migration time), internal normalization standards (green arrow) maltose (9.86) and cellooctaose (13.92). Synthesis of compound **18** (red, lacto-*N*-neo-octaose, Gal(β1,4)GlcNAc(β1,3)Gal(β1,4) GlcNAc(β1,3)Gal(β1,4)GlcNAc(β1,3)Gal(β1,4)Glc) and compound **24** (blue, lacto-*N*-octaose, Gal(β1,3)GlcNAc(β1,3)Gal(β1,4)GlcNAc(β1,3)Gal(β1,4)GlcNAc(β1,3)Gal(β1,4)Glc).

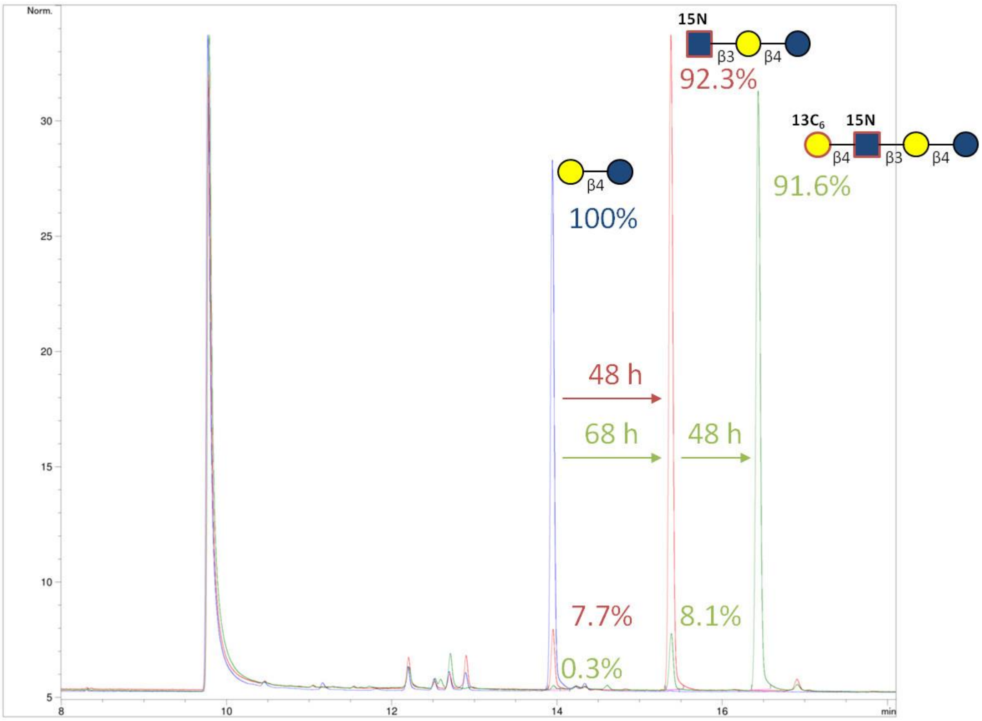


**Figure S6.** CE-LIF overlay of the reaction monitoring after respective 48 hours, (normalized migration time) EOF (9.86), maltononaose (not shown), sequential synthesis of [$^{15}$N/$^{13}$C$_6$]lacto-*N*-neo-tetraose, [$^{13}$C$_6$]Gal(β1,4)[$^{15}$N]GlcNAc(β1,3)Gal(β1,4)Glc. The total yield was calculated from the relative conversion observed within the final analysis (green).

**Mass spectrometry (ESI-MS)**

Regarding the product evaluation, we have to notice that some ESI-MS spectra for the linear long-chained structures show an unfavorable signal noise to ratio. Therefore, the more sensitive and more high-grade MALDI-TOF-MS measurements of the same samples were additionally taken into account. The unfavorable signal noise to ratio of the ESI-MS HMOS analysis is may caused by the challenging isomerization properties of the analyzed linear long-chained structures which is crucially improved by the MALDI-TOF-MS technique. Critical ESI-MS spectra were supplemented with spectra scans.

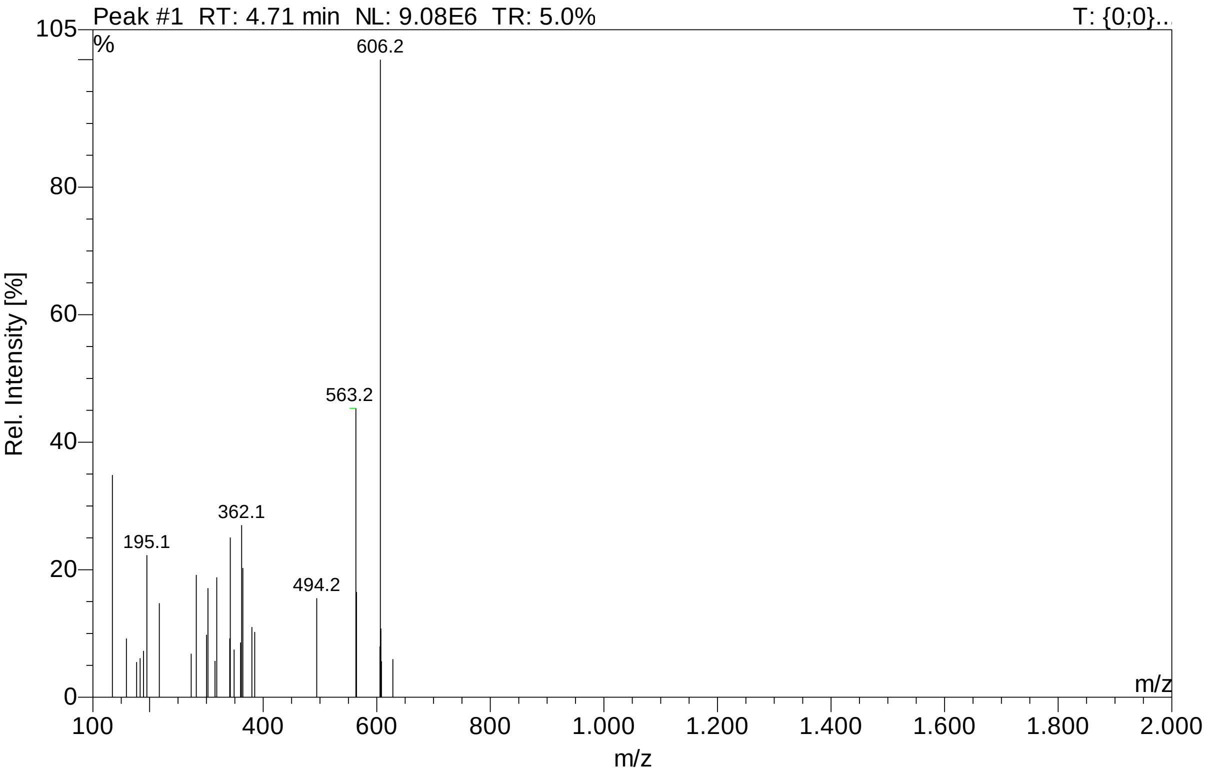


**Figure MS1.** Mass spectrum (ESI-) of UDP-*N*-acetyl-glucosamine ([M-H]-, *m/z* 606.2).

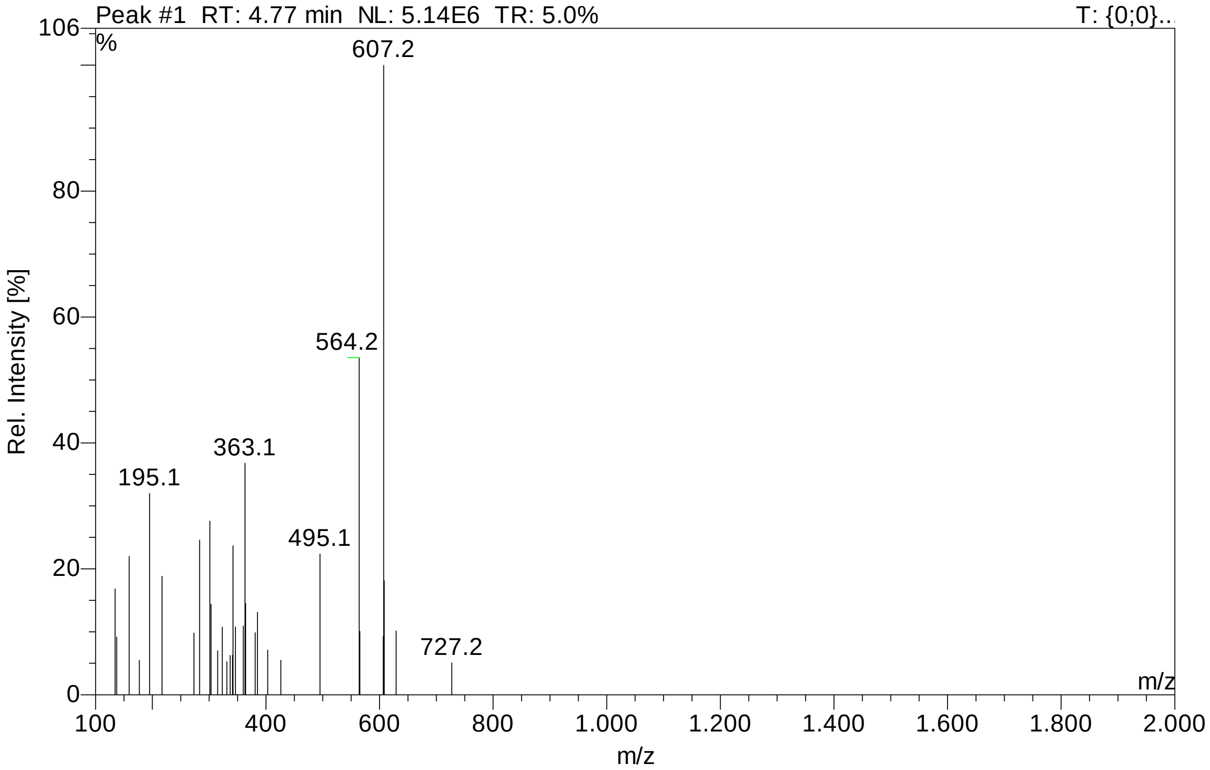


**Figure MS2.** Mass spectrum (ESI-) of UDP-*N*-acetyl-[$^{15}$N]glucosamine (compound **6**) ([M-H]$^-$, *m/z* 607.2).

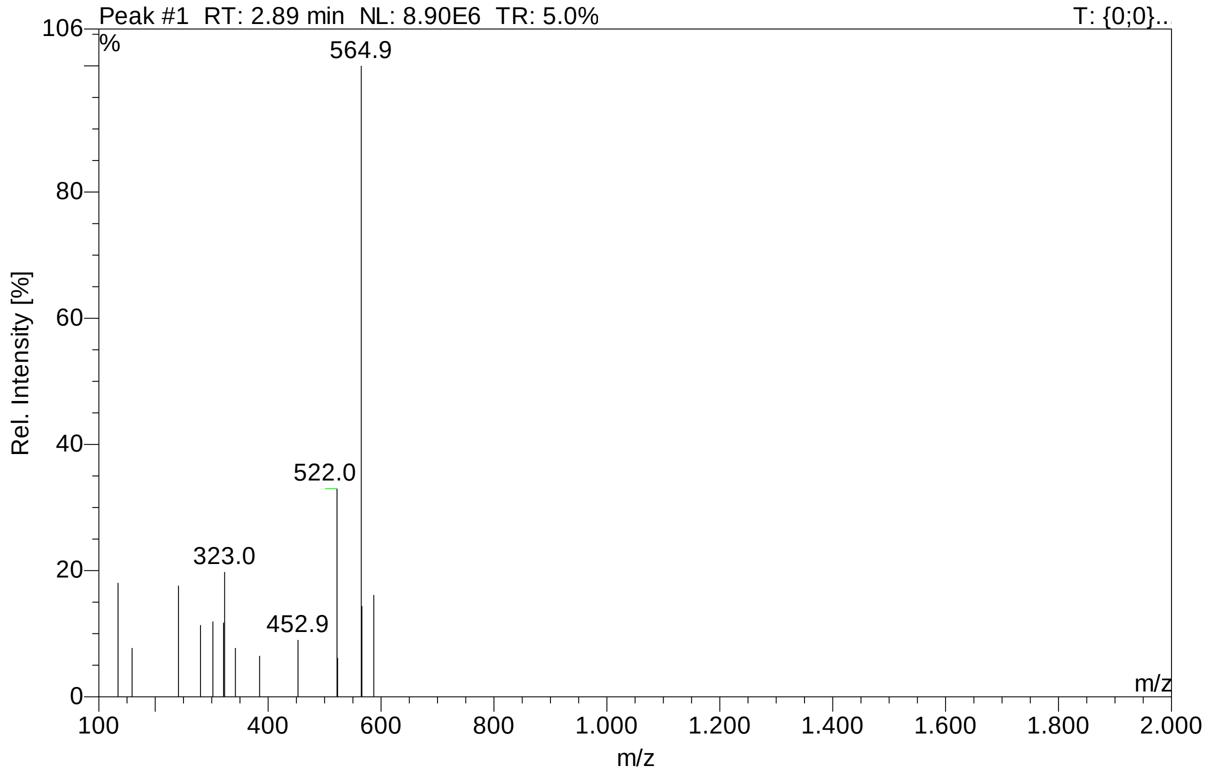


**Figure MS3.** Mass spectrum (ESI-) of UDP-galactose ([M-H]$^-$, *m/z* 564.9).

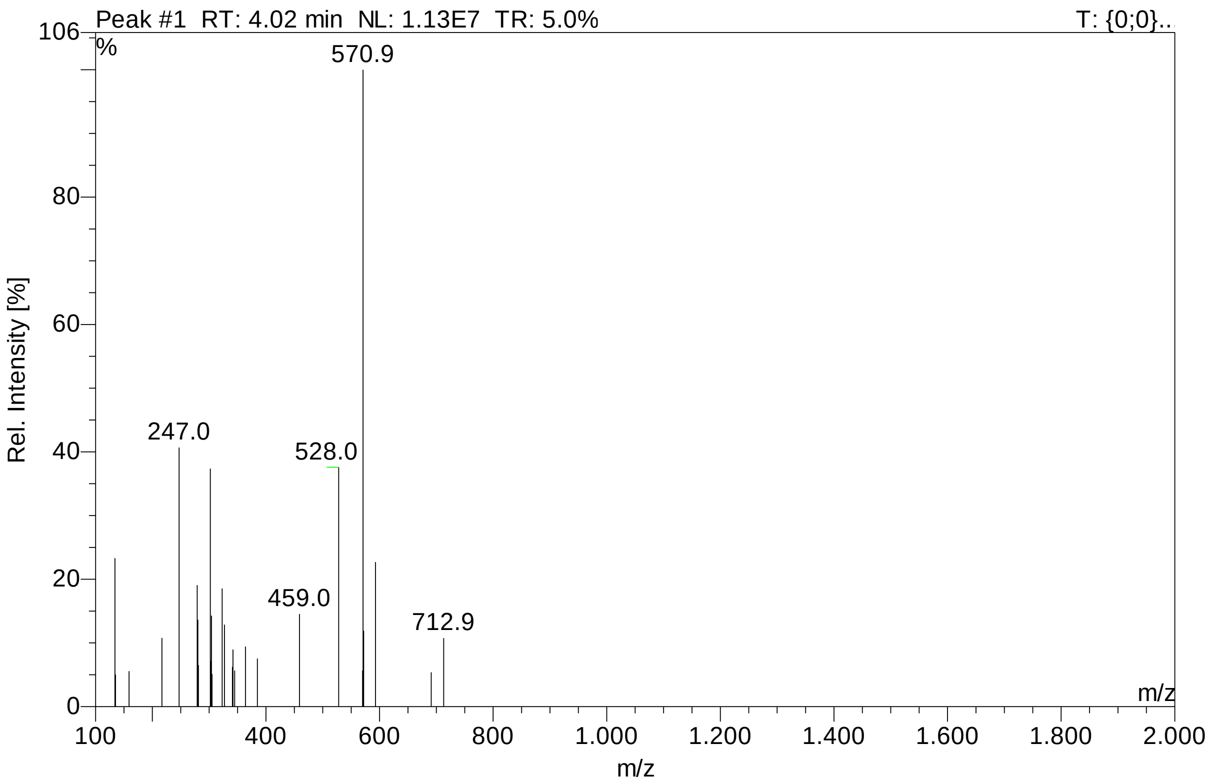


**Figure MS4**. Mass spectrum (ESI-) of UDP-[$^{13}C_6$]galactose **3** ($[M-H]^-$, *m/z* 570.9).

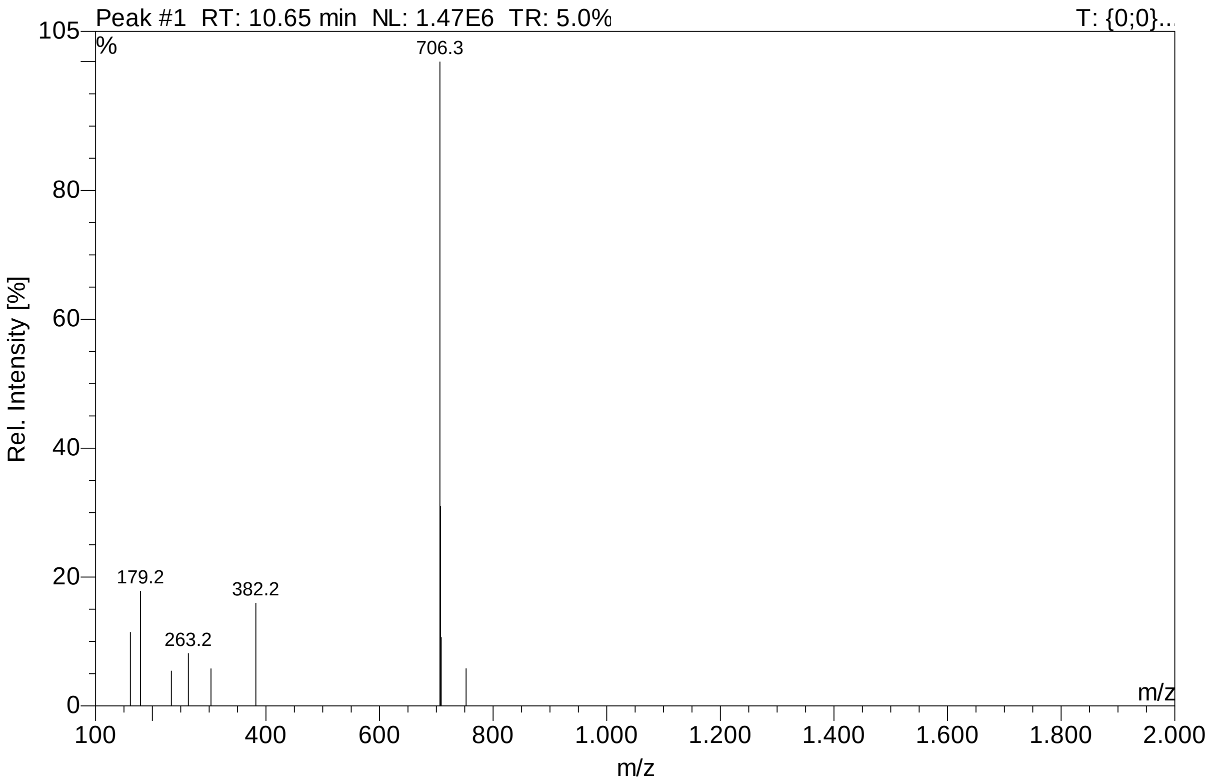


**Figure MS5.** Mass spectrum (ESI-) of lacto-*N*-tetraose **19**, Gal(β1,3)GlcNAc(β1,3)Gal(β1,4)Glc ($[M-H]^-$, *m/z* 706.3).

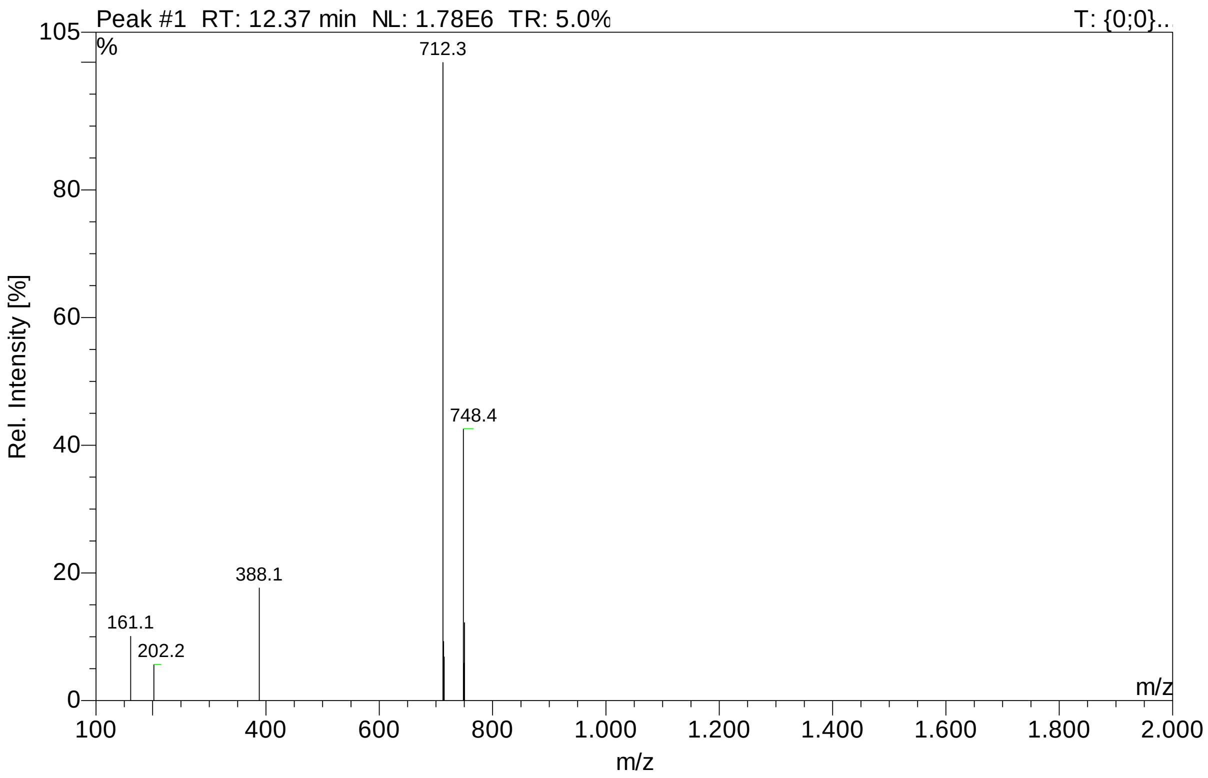


**Figure MS6.** Mass spectrum (ESI-) of [$^{13}C_6$]lacto-*N*-tetraose **20**, [$^{13}C_6$]Gal(β1,3)GlcNAc(β1,3)Gal(β1,4)Glc ([M-H]⁻, *m/z* 712.3; [M+Cl]⁻, *m/z* 748.4).

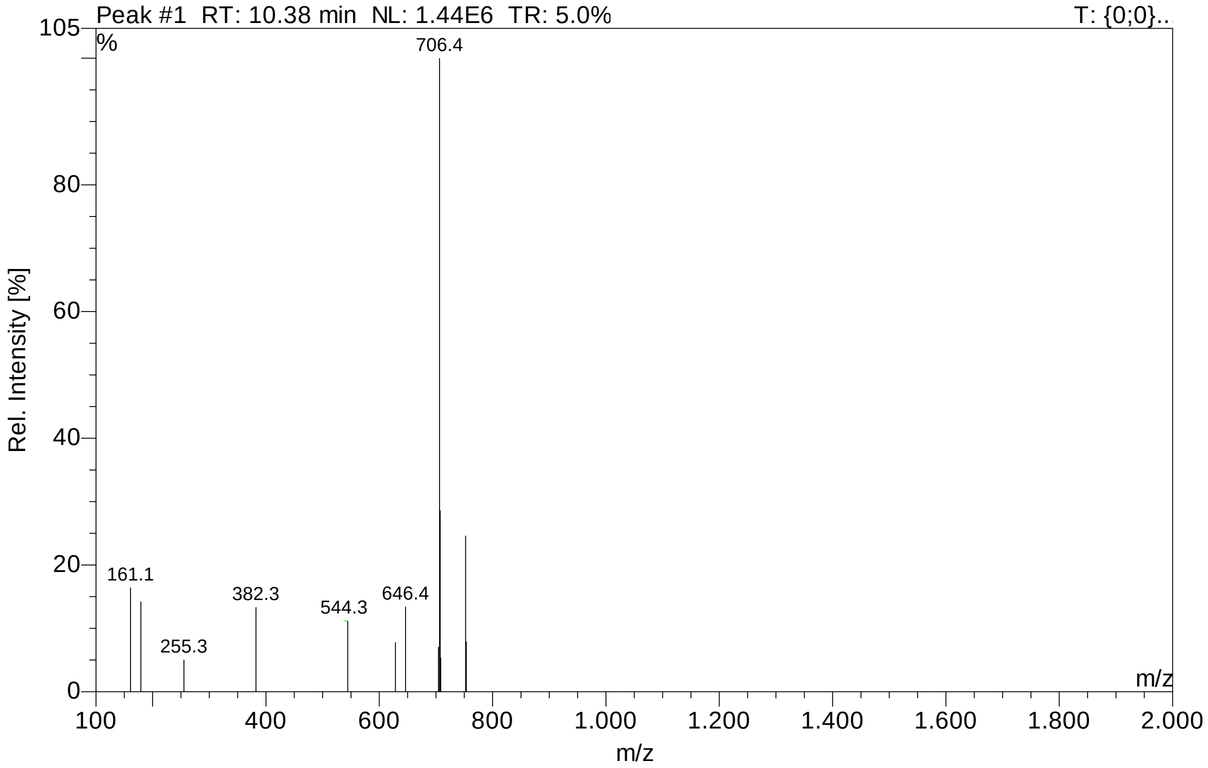


**Figure MS7.** Mass spectrum (ESI-) of lacto-*N*-neo-tetraose **9**, Gal(β1,4)GlcNAc(β1,3)Gal(β1,4)Glc ([M-H]⁻, *m/z* 706.4).

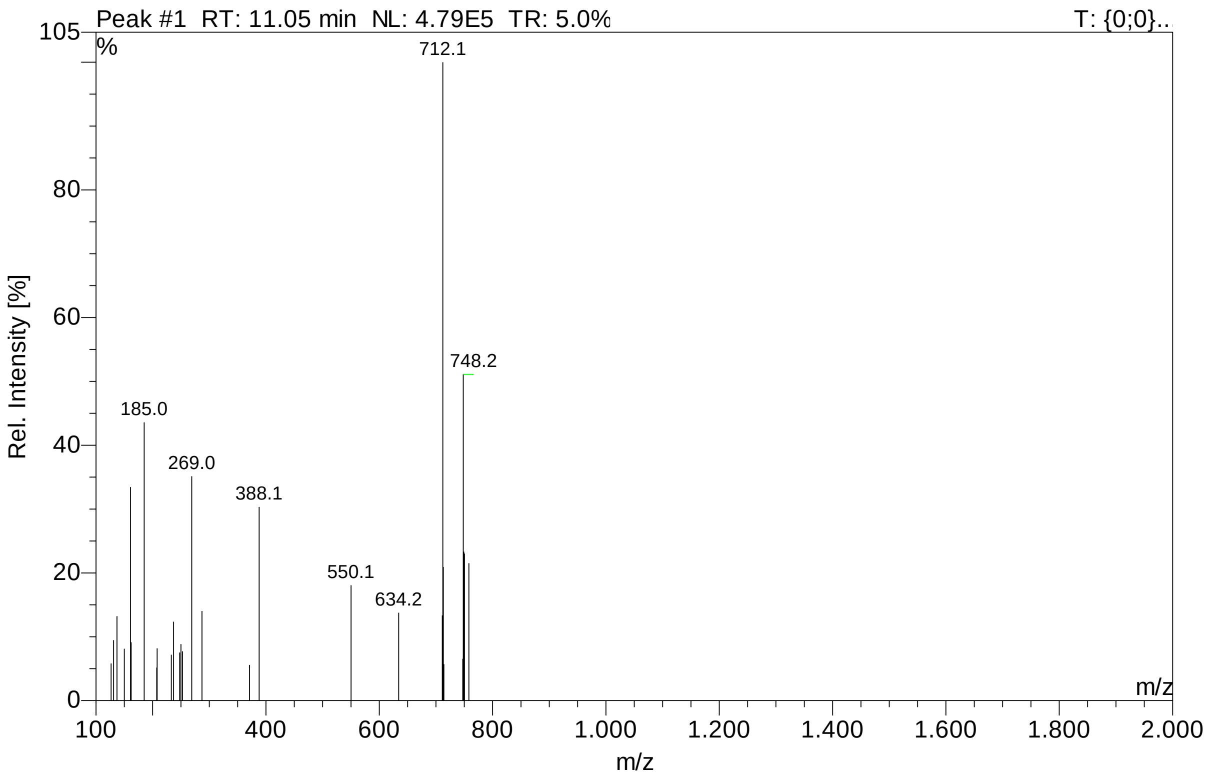


**Figure MS8.** Mass spectrum (ESI-) of [$^{13}$C6]lacto-*N*-neo-tetraose **10**, [$^{13}$C6]Gal(β1,4)GlcNAc (β1,3)Gal(β1,4)Glc ([M-H]-, *m/z* 712.1; [M+Cl]-, *m/z* 748.2).

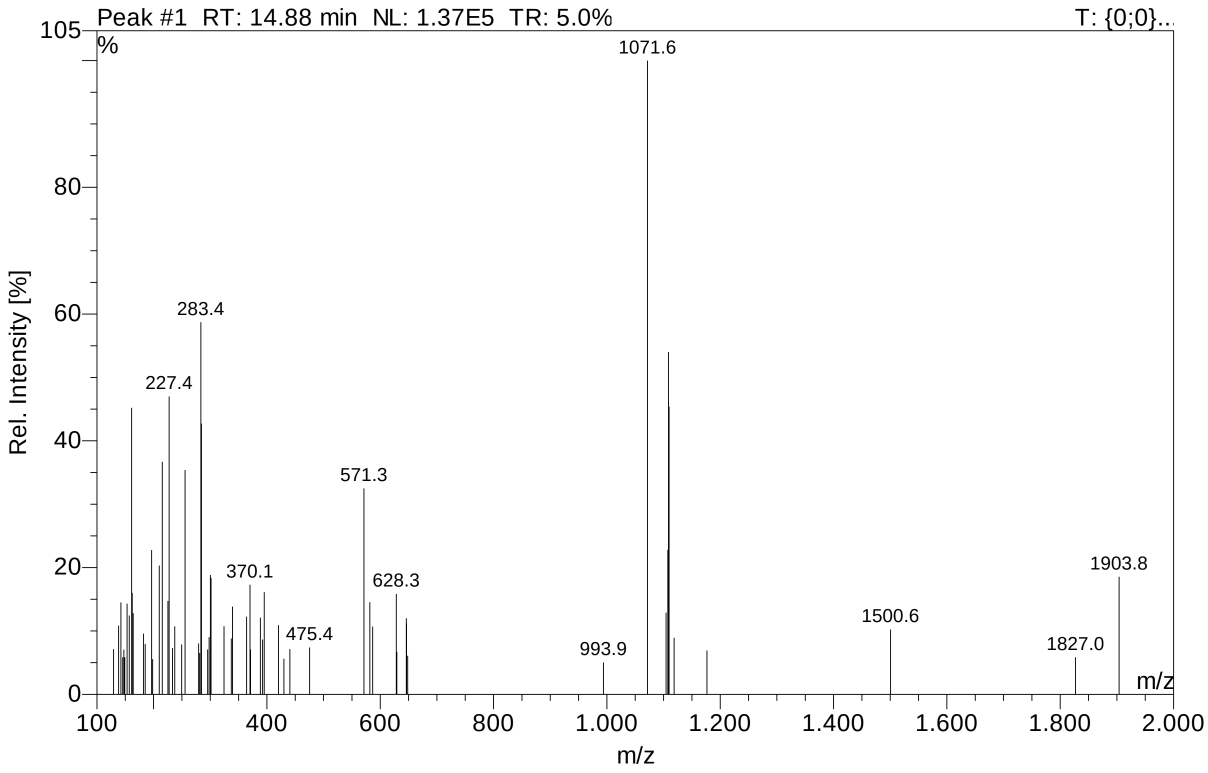


**Figure MS9.** Mass spectrum (ESI-) of lacto-*N*-hexaose **21**, Gal(β1,3)GlcNAc(β1,3)Gal(β1,4) GlcNAc(β1,3)Gal(β1,4)Glc ([M-H]-, *m/z* 1071.6).

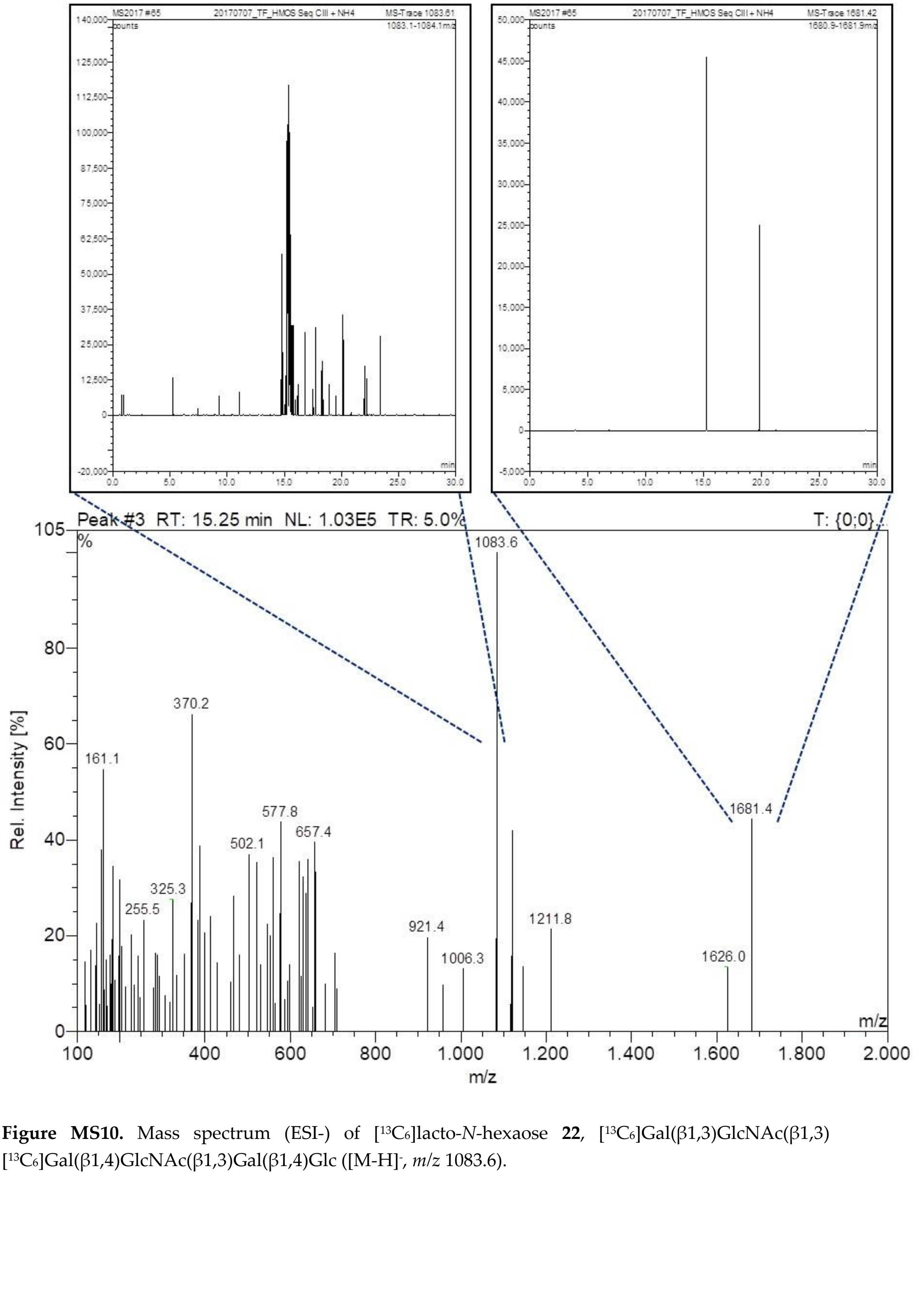


**Figure MS10.** Mass spectrum (ESI-) of [$^{13}C_6$]lacto-*N*-hexaose **22**, [$^{13}C_6$]Gal(β1,3)GlcNAc(β1,3)[$^{13}C_6$]Gal(β1,4)GlcNAc(β1,3)Gal(β1,4)Glc ([M-H]$^-$, *m/z* 1083.6).

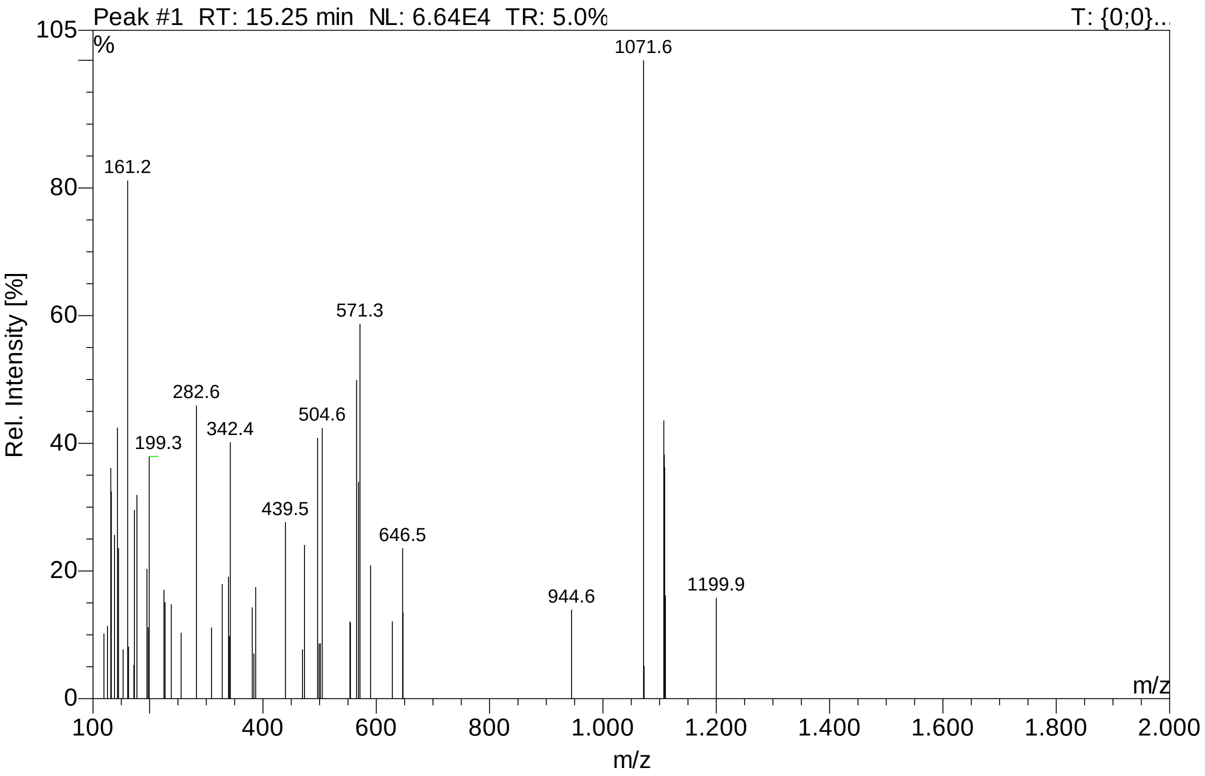


**Figure MS11.** Mass spectrum (ESI-) of lacto-*N*-neo-hexaose **13**, Gal(β1,4)GlcNAc(β1,3)Gal(β1,4) GlcNAc(β1,3)Gal(β1,4)Glc ([M-H]$^-$, *m/z* 1071.6).

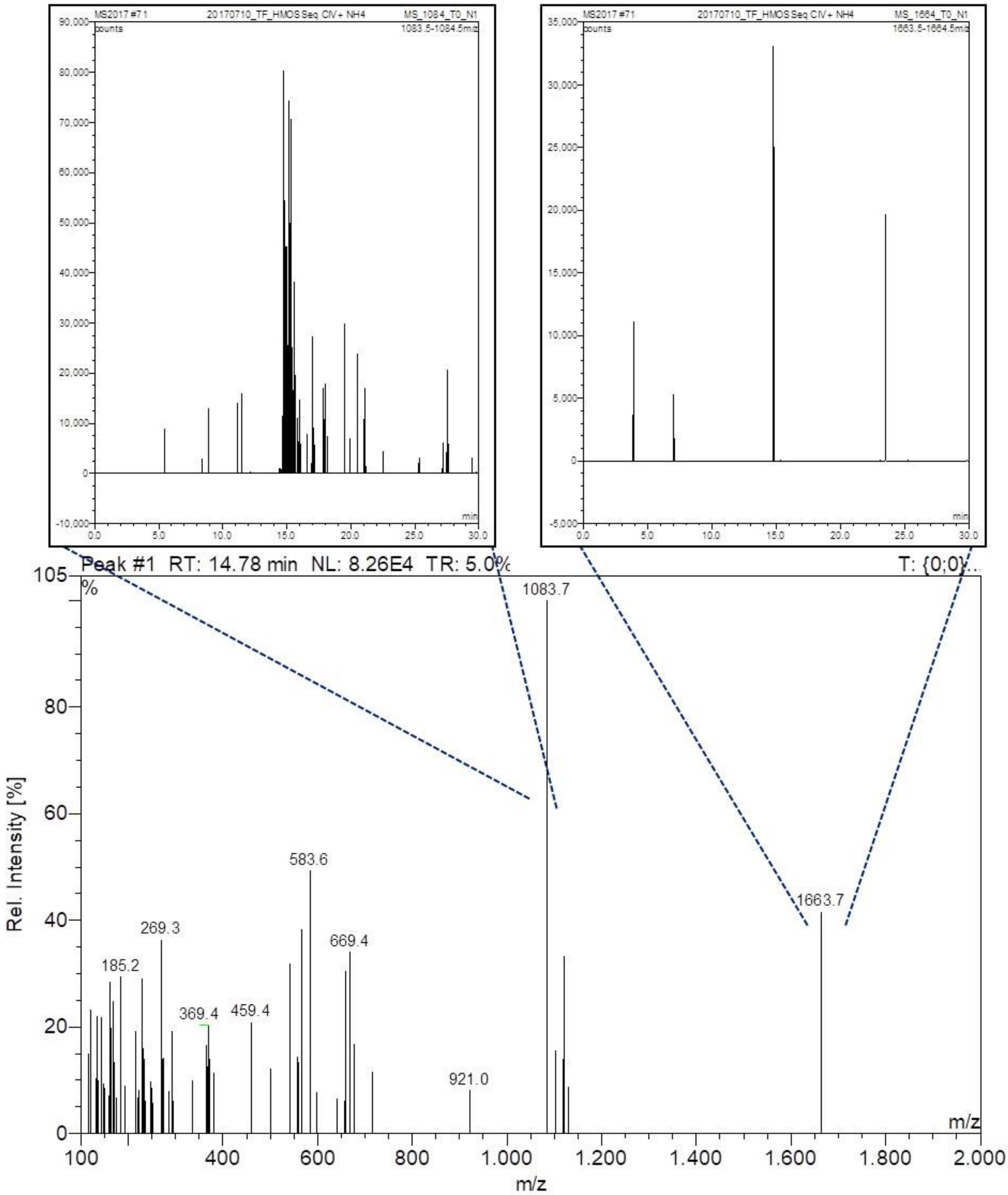


**Figure MS12.** Mass spectrum (ESI-) of [$^{13}C_6$]lacto-*N*-neo-hexaose **14**, [$^{13}C_6$]Gal(β1,4)GlcNAc(β1,3)[$^{13}C_6$]Gal(β1,4)GlcNAc(β1,3)Gal(β1,4)Glc ([M-H]$^-$, *m/z* 1083.7).

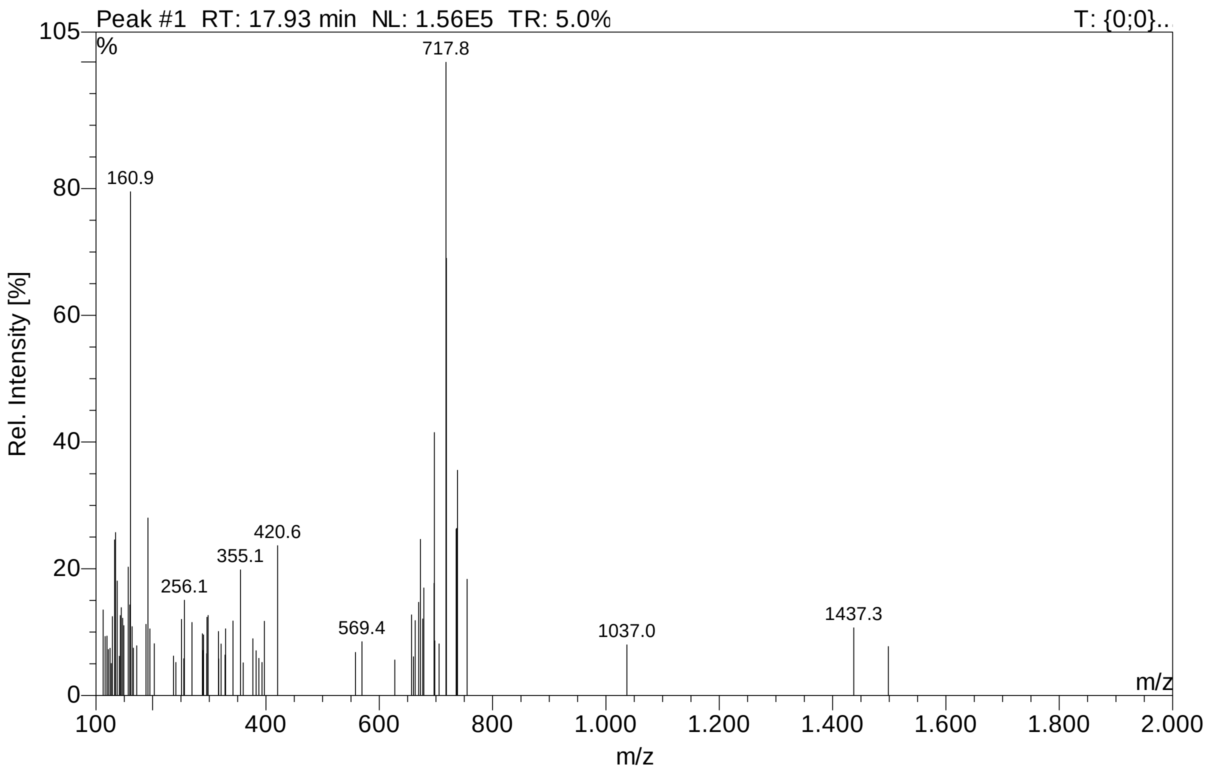


**Figure MS13.** Mass spectrum (ESI-) of lacto-*N*-octaose **23**, Gal(β1,3)GlcNAc(β1,3)Gal(β1,4) GlcNAc(β1,3)Gal(β1,4)GlcNAc(β1,3)Gal(β1,4)Glc ([M-2H]-, *m/z* 717.8, [M-H]-, *m/z* 1437.3).

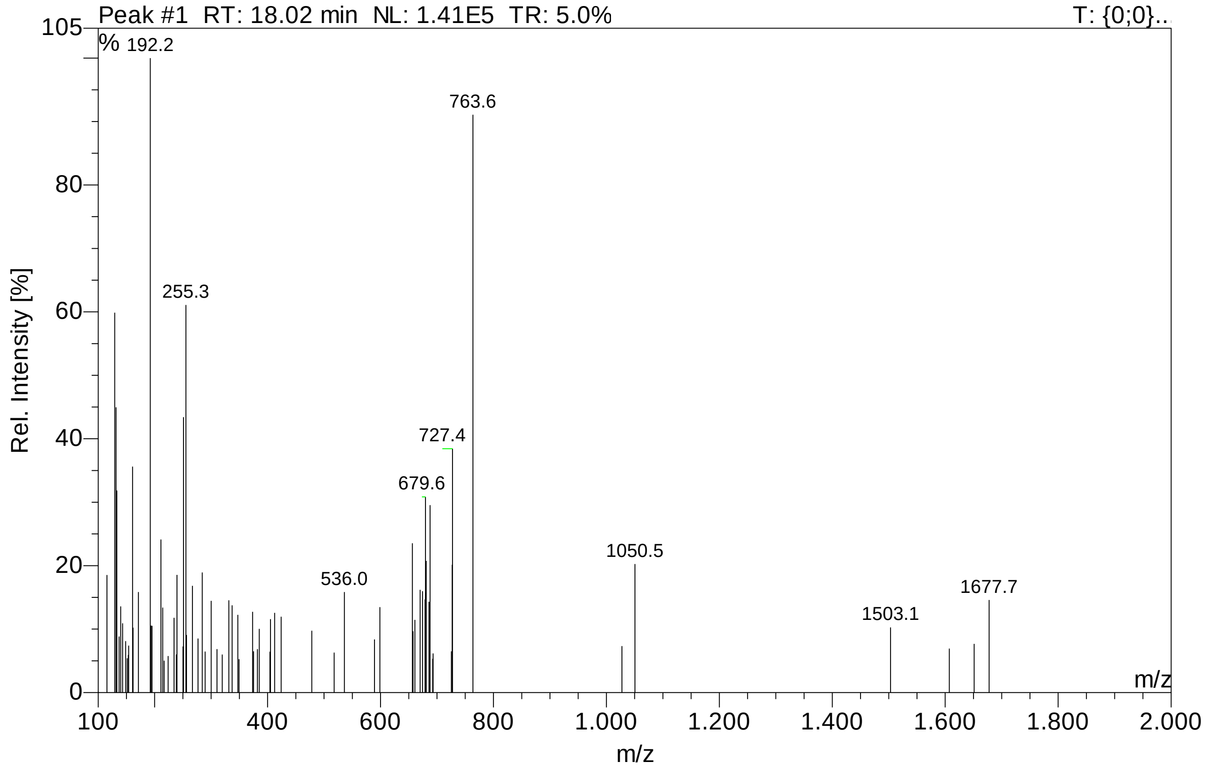


**Figure MS14.** Mass spectrum (ESI-) of [$^{13}C_6$]lacto-*N*-octaose **24**, [$^{13}C_6$]Gal(β1,3)GlcNAc(β1,3) [$^{13}C_6$]Gal(β1,4)GlcNAc(β1,3)[$^{13}C_6$]Gal(β1,4)GlcNAc(β1,3)Gal(β1,4)Glc ([M-2H]-, *m/z* 727.1, [M-2H+Cl]- , *m/z* 748.4).

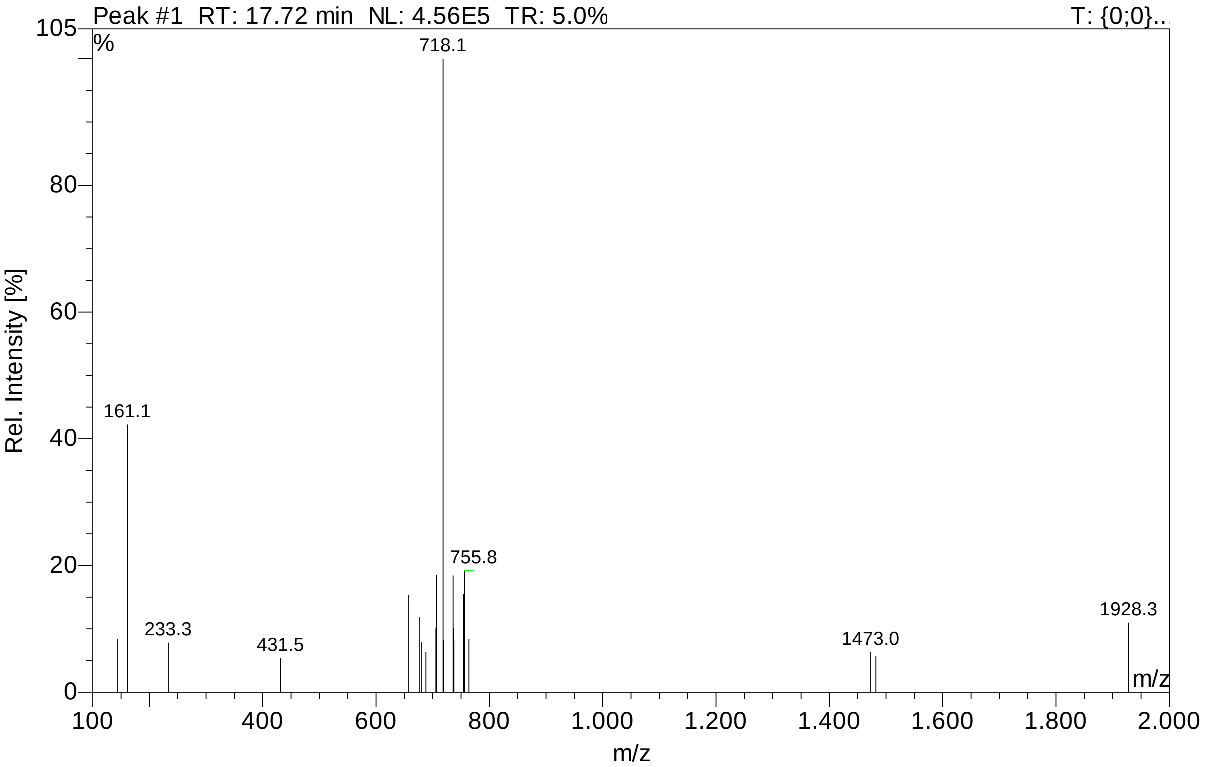


**Figure MS15.** Mass spectrum (ESI-) of lacto-*N*-neo-octaose **17**, [$^{13}C_6$]Gal(β1,4)GlcNAc(β1,3)[$^{13}C_6$]Gal(β1,4)GlcNAc(β1,3)[$^{13}C_6$]Gal(β1,4)GlcNAc(β1,3)Gal(β1,4)Glc ([M-2H]$^-$, *m/z* 718.1, [M+K-2H]$^-$, *m/z* 1473.0).

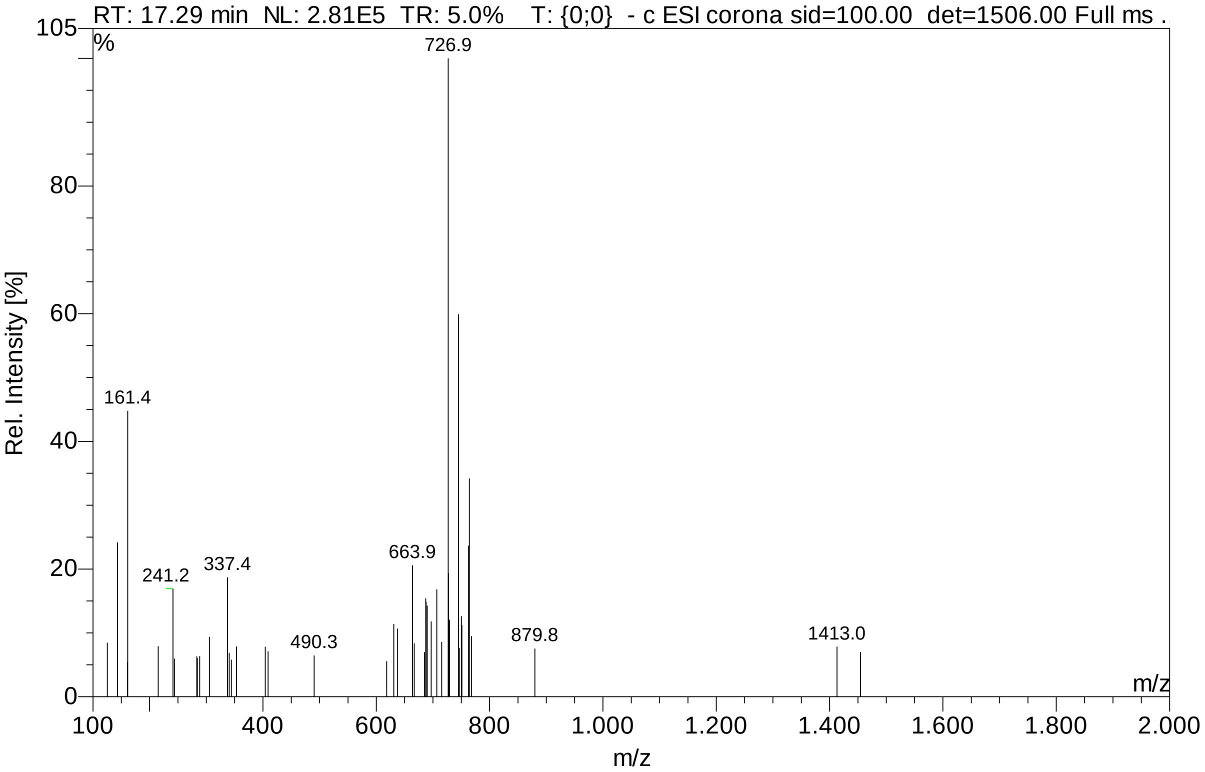


**Figure MS16.** Mass spectrum (ESI-) of [$^{13}C_6$]lacto-*N*-neo-octaose **18**, [$^{13}C_6$]Gal(β1,4)GlcNAc(β1,3)[$^{13}C_6$]Gal(β1,4)GlcNAc(β1,3)[$^{13}C_6$]Gal(β1,4)GlcNAc(β1,3)Gal(β1,4)Glc ([M-2H]$^-$, *m/z* 726.9).

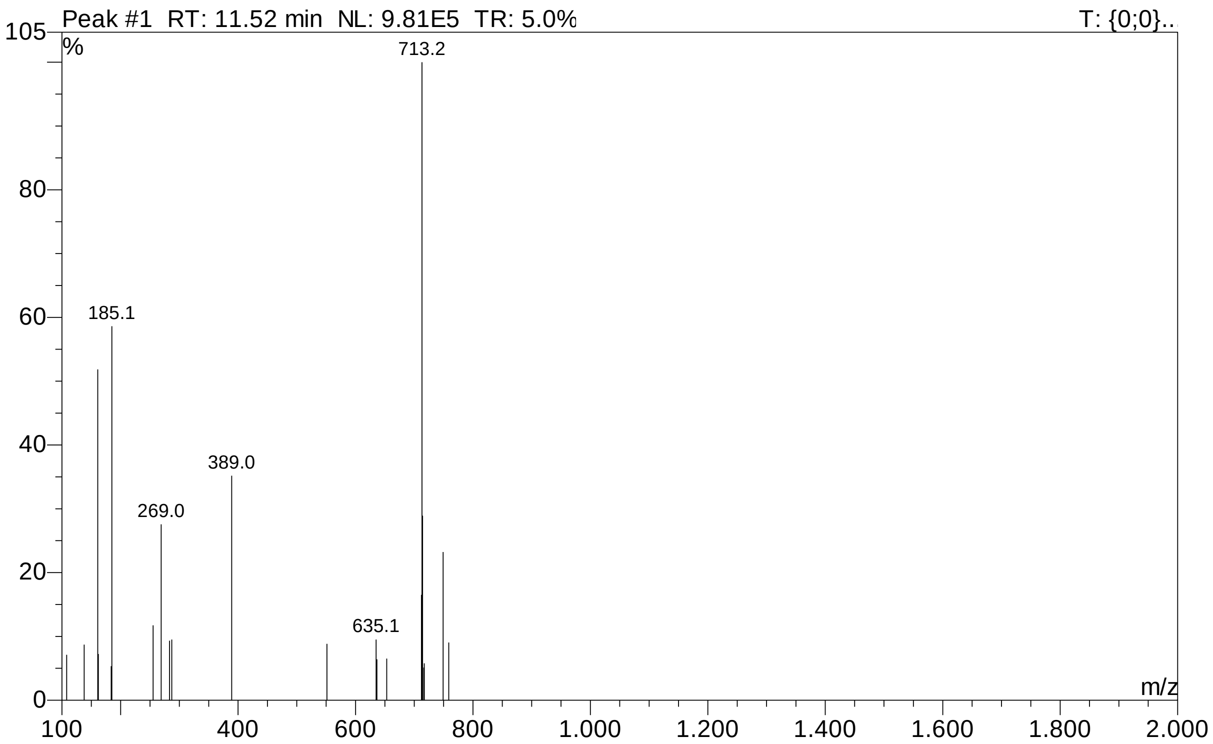


**Figure MS17.** Mass spectrum (ESI-) of [$^{15}$N/$^{13}$C$_6$]lacto-*N*-neo-tetraose **25**, [$^{13}$C$_6$]Gal(β1,4)[$^{15}$N]GlcNAc(β1,3)Gal(β1,4)Glc ([M-2H]$^-$, *m/z* 713.2).

## Mass spectrometry (MALDI-TOF-MS)

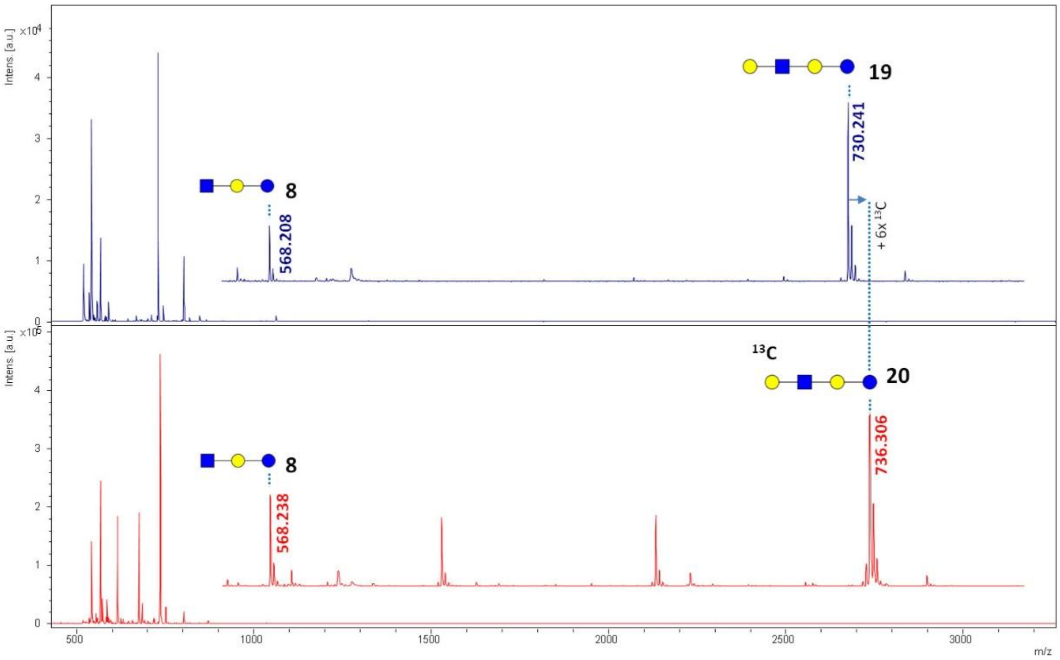


**Figure MTS1.** Mass spectrum (MALDI-TOF-MS) of compound **8** lacto-*N*-triaose II, GlcNAc(β1,3)Gal(β1,4)Glc ([M+Na]$^+$, *m*/*z* 568.2), synthesis product **19** lacto-*N*-tetraose, Gal(β1,3)GlcNAc(β1,3)Gal(β1,4)Glc ([M+Na]$^+$, *m*/*z* 730.2) and **20** [$^{13}C_6$]lacto-*N*-tetraose, [$^{13}C_6$]Gal(β1,3)GlcNAc(β1,3)Gal(β1,4)Glc ([M+Na] $^+$, *m*/*z* 736.3).

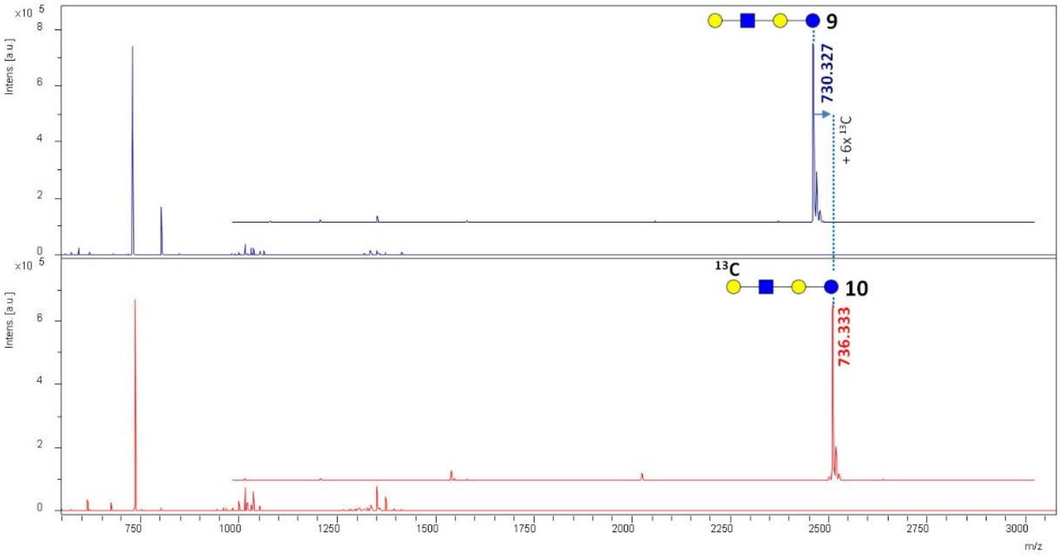


**Figure MTS2.** Mass spectrum (MALDI-TOF-MS) of compound **9** lacto-*N*-neo-tetraose, Gal(β1,4)GlcNAc(β1,3)Gal(β1,4)Glc ([M+Na]$^+$, *m*/*z* 730.2) and **10** [$^{13}C_6$]lacto-*N*-neo-tetraose, [$^{13}C_6$]Gal(β1,4)GlcNAc(β1,3)Gal(β1,4)Glc ([M+Na]$^+$, *m*/*z* 736.3).

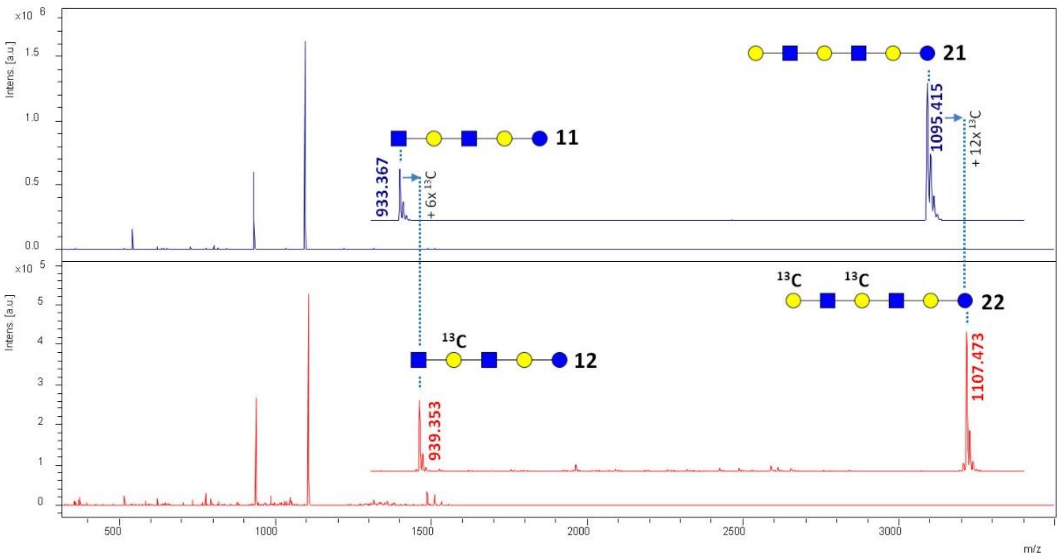


**Figure MTS3.** Mass spectrum (MALDI-TOF-MS) of compound **11** lacto-*N*-pentaose, GlcNAc(β1,3)Gal(β1,4)GlcNAc(β1,3)Gal(β1,4)Glc ([M+Na]+, *m/z* 933.4), **12** [$^{13}C_6$]lacto-*N*-pentaose, GlcNAc(β1,3)[$^{13}C_6$]Gal(β1,4)GlcNAc(β1,3)Gal(β1,4)Glc ([M+Na]+, *m/z* 939.4), **21** lacto-*N*-hexaose, Gal(β1,3)GlcNAc(β1,3)Gal(β1,4)GlcNAc(β1,3)Gal(β1,4)Glc ([M+Na]+, *m/z* 1095.4) and **22** [$^{13}C_6$]lacto-*N*-hexaose, [$^{13}C_6$]Gal(β1,3)GlcNAc(β1,3)[$^{13}C_6$]Gal(β1,4)GlcNAc(β1,3)Gal(β1,4)Glc ([M+Na]+, *m/z* 1107.5).

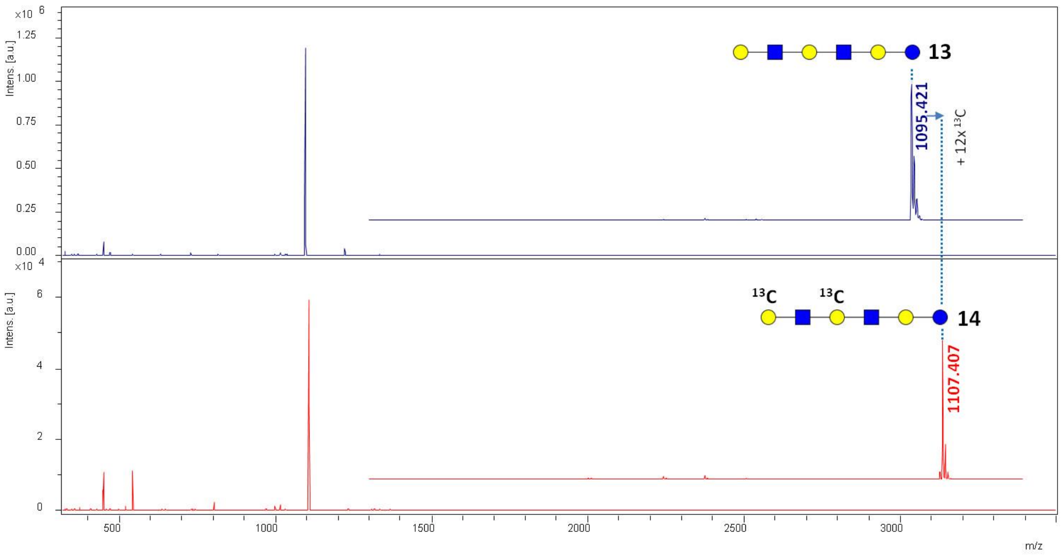


**Figure MTS4.** Mass spectrum (MALDI-TOF-MS) of compound **13** lacto-*N*-neo-hexaose, Gal(β1,4)GlcNAc(β1,3)Gal(β1,4)GlcNAc(β1,3)Gal(β1,4)Glc ([M+Na]+, *m/z* 1095.4) and **14** [$^{13}C_6$]lacto-*N*-neo-hexaose, [$^{13}C_6$]Gal(β1,4)GlcNAc(β1,3)[$^{13}C_6$]Gal(β1,4)GlcNAc(β1,3)Gal(β1,4)Glc ([M+Na]+, *m/z* 1107.4).

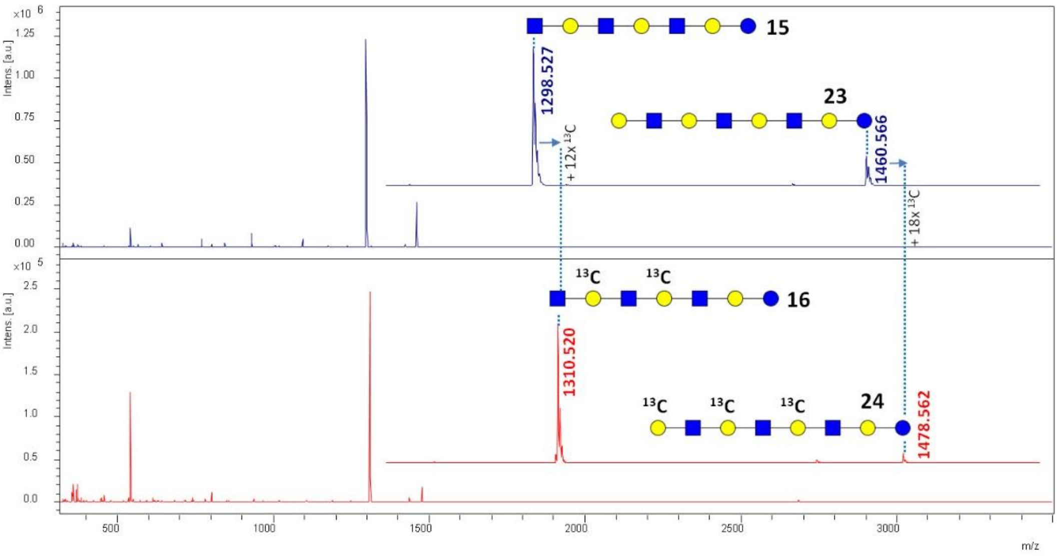


**Figure MTS5.** Mass spectrum (MALDI-TOF-MS) of compound **15** lacto-*N*-heptaose, GlcNAc(β1,3)Gal(β1,4)GlcNAc(β1,3)Gal(β1,4)GlcNAc(β1,3)Gal(β1,4)Glc ([M+Na]$^+$, *m*/*z* 1298.5), **16** [$^{13}C_6$]lacto-*N*-heptaose, GlcNAc(β1,3)[$^{13}C_6$]Gal(β1,4)GlcNAc(β1,3)[$^{13}C_6$]Gal(β1,4)GlcNAc(β1,3) Gal(β1,4)Glc ([M+Na]$^+$, *m*/*z* 1310.5), **23** lacto-*N*-octaose, Gal(β1,3)GlcNAc(β1,3)Gal(β1,4) GlcNAc(β1,3)Gal(β1,4)GlcNAc(β1,3)Gal(β1,4)Glc ([M+Na]$^+$, *m*/*z* 1460.6) and **24** [$^{13}C_6$]lacto-*N*-octaose, [$^{13}C_6$]Gal(β1,3)GlcNAc(β1,3)[$^{13}C_6$]Gal(β1,4)GlcNAc(β1,3)[$^{13}C_6$]Gal(β1,4)GlcNAc(β1,3)Gal(β1,4)Glc ([M+Na]$^+$, *m*/*z* 1478.6).

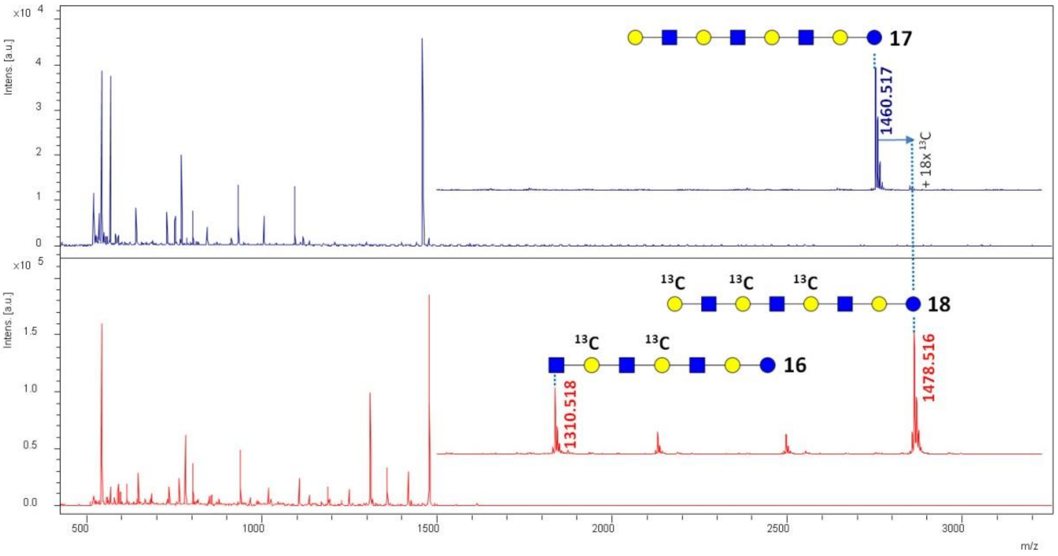


**Figure MTS6.** Mass spectrum (MALDI-TOF-MS) of compound **16** [$^{13}C_6$]lacto-*N*-heptaose, GlcNAc(β1,3)[$^{13}C_6$]Gal(β1,4)GlcNAc(β1,3)[$^{13}C_6$]Gal(β1,4)GlcNAc(β1,3)Gal(β1,4)Glc ([M+Na]$^+$, *m*/*z* 1310.5), **17** lacto-*N*-neo-octaose, Gal(β1,4)GlcNAc(β1,3)Gal(β1,4)GlcNAc(β1,3)Gal(β1,4) GlcNAc(β1,3)Gal(β1,4)Glc ([M+Na]$^+$, *m*/*z* 1460.5) and **18** [$^{13}C_6$]lacto-*N*-octaose, [$^{13}C_6$]Gal(β1,4) GlcNAc(β1,3)[$^{13}C_6$]Gal(β1,4)GlcNAc(β1,3)[$^{13}C_6$]Gal(β1,4)GlcNAc(β1,3)Gal(β1,4)Glc ([M+Na]$^+$, *m*/*z* 1478.5).

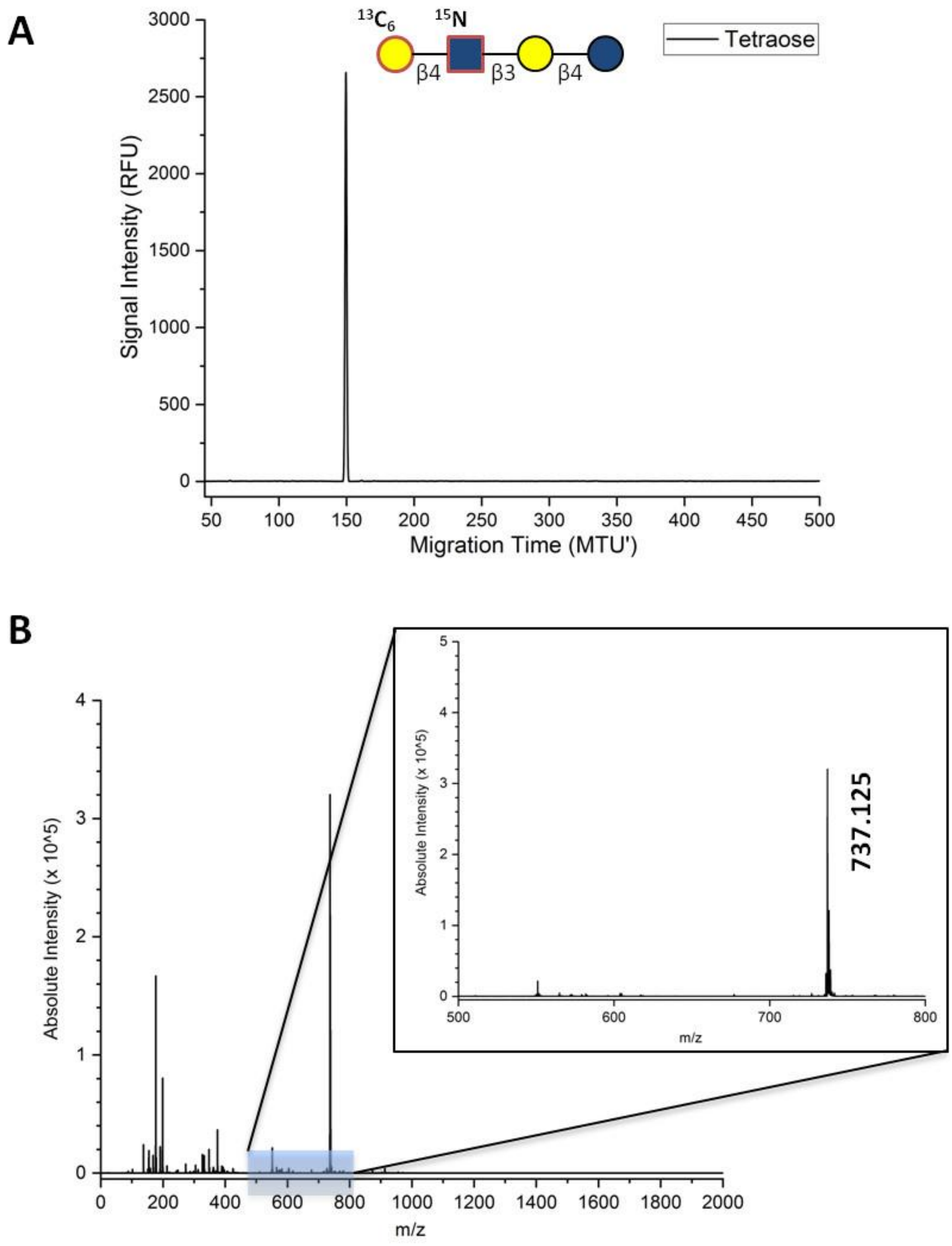


**Figure MTS7.** (**A**) xCGE-LIF analysis of the purified [$^{15}$N/$^{13}$C$_6$]lacto-*N*-neo-tetraose, (**B**) mass spectrum (MALDI-TOF-MS) of the purified compound **25** [$^{13}$C$_6$/$^{15}$N]lacto-*N*-neo-tetraose, [$^{13}$C$_6$]Gal(β1,4)[$^{15}$N]GlcNAc(β1,3)Gal(β1,4)Glc ([M+Na]$^+$, *m*/*z* 737.1).

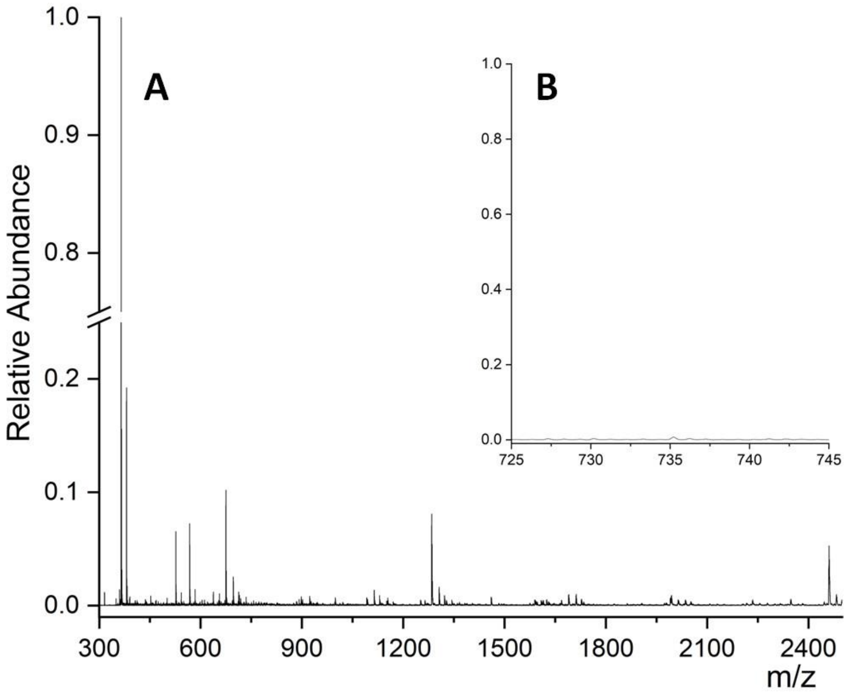


**Figure MTS8.** MALDI-TOF mass spectrum of PGC-SPE extracted bovine milk (**A**) and the magnified *m*/*z* range from 726 to 746 (**B**).

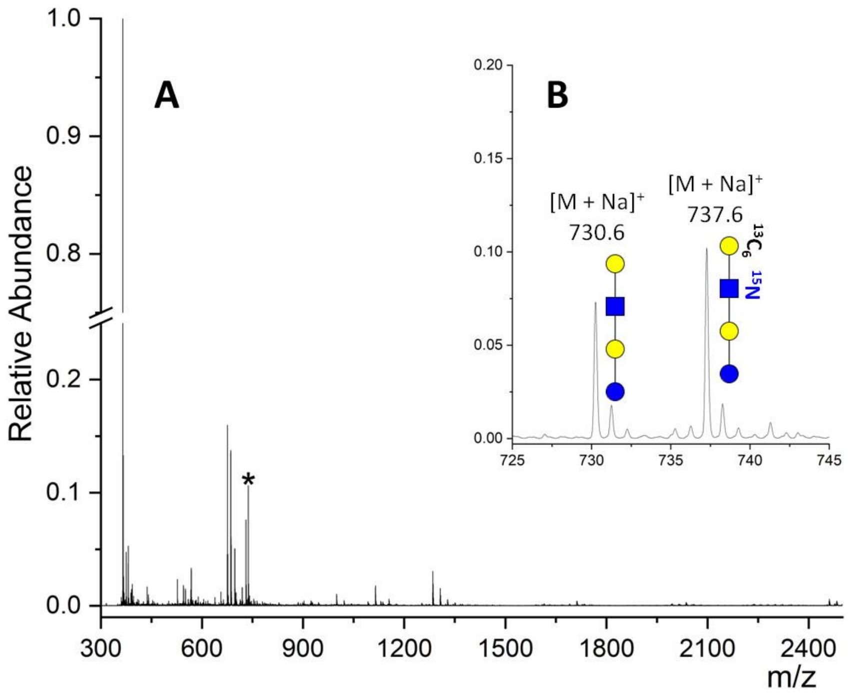


**Figure MTS9.** MALDI-TOF mass spectrum of PGC-SPE extracted bovine milk spiked with LNnT and [$^{13}C_6$/$^{15}N$]LNnT in the *m*/*z* range 300 to 2500 (**A**) and a zoom into the *m*/*z* range 725 to 745 (**B**). An asterisk indicates the signals corresponding to LNnT (*m*/*z* = 730.6204 [M+Na]$^+$) and [$^{13}C_6$/$^{15}N$]LNnT (*m*/*z* = 737.641 [M+Na]$^+$).

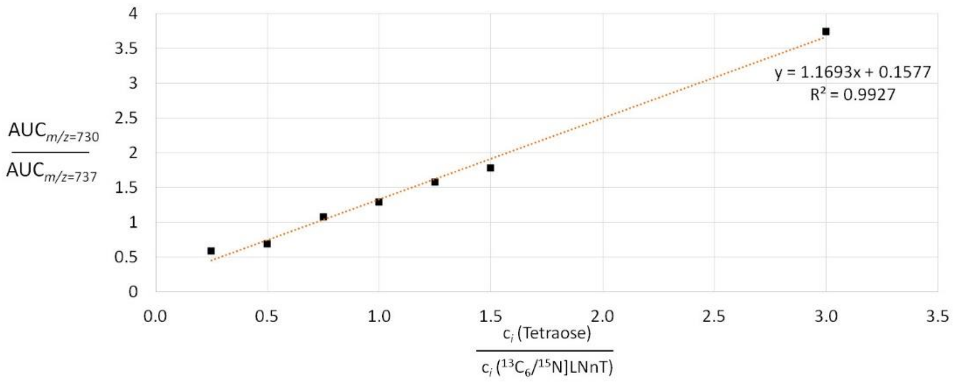


**Figure S7.** Calibration plot for the response function correlating relative changes of the AUC to varying molar ratios of tetraose to [$^{13}C_6$/$^{15}N$]LNnT. 1 µL of [$^{13}C_6$/$^{15}N$]LNnT (1 mM) was mixed with human milk (0.1 to 3 µL). The graph displays averaged triplicates with error bars representing the standard deviation.

**Purity determination via TOC analysis**

According to the xCGE-LIF analysis, the purified [$^{15}N$/$^{13}C_6$]LNnT sample is broadly free from other sugar structures, accessible for APTS labelling, like the remaining synthesis substrates and intermediates. This indicates smaller synthesis compounds were widely removed during the ultra-dialysis (cut-off - 100-500 Da, FloatALyser® G2). To complete the product evaluation, a TOC analysis was implemented into the analytical workflow. Presuming the purified [$^{15}N$/$^{13}C_6$]LNnT sample is largely free of miscellaneous organic carbon allows us to calculate a sample purity of 96.05% based on the TOC data.

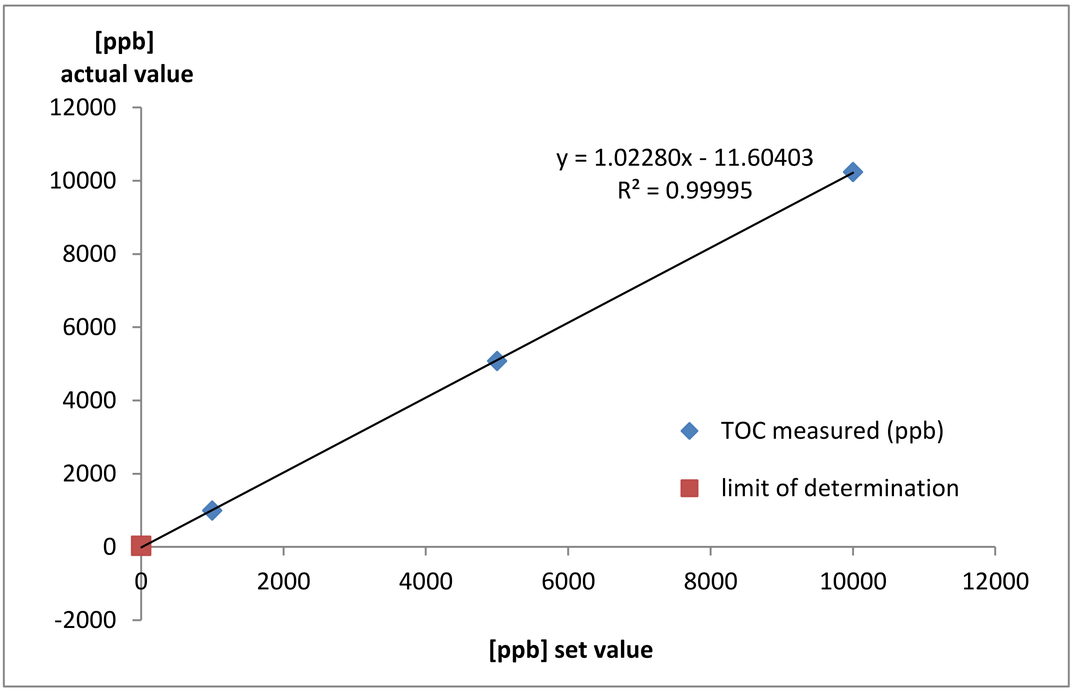


**Figure TOCS1.** Initial calibration with potassium hydrogen phthalate. The limit of determination was determined to 4.1 ppb, the limit of quantification to 12.3 ppb and the $R^2$ of the initial calibration features 0.99995.

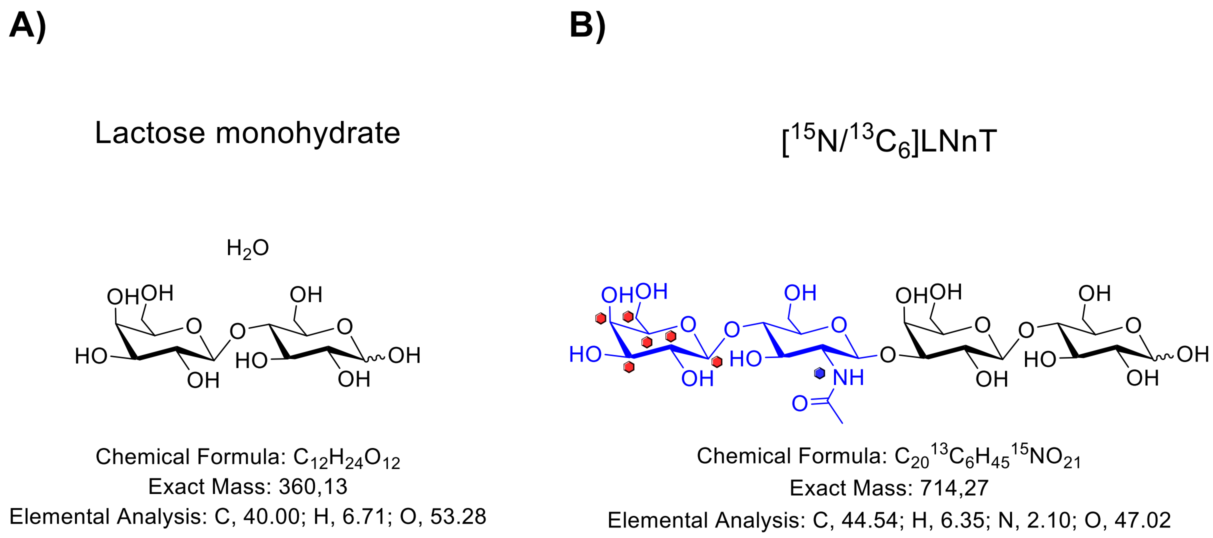


**Scheme TOCS1.** Calculated exact mass and elemental mass percentage for(**A**) Lactose monohydrate and (**B**) $[^{15}N/^{13}C_6]$LNnT.

**Table TOCS1.** Measured TOC values and carbon mass percentage of highly pure commercial references substances as well as from the $[^{15}N/^{13}C_6]$LNnT sample.

| **Sample** | **Sample amount [mg]** | **Sample volume [mL]** | **TOC (ppb)** | **TOC [µg]** | **Carbon Mass Percentage [m-%]** |
|---|---|---|---|---|---|
| **Lactose monohydrate, (99,5%)** | 1.78 | 17.8 | 40053 | 712.9 | 40.23 |
| | | | 40401 | 719.1 | |
| **Lactose monohydrate, Sigma** | 1.27 | 12.7 | 39205 | 497.9 | 39.09 |
| | | | 38967 | 494.9 | |
| **Maltose monohydrate, Supelco** | 1.25 | 12.5 | 40248 | 503.1 | 40.29 |
| | | | 40336 | 504.2 | |
| **$[^{15}N/^{13}C_6]$LNnT** | 0.05 | 9 | 2576 | 23.2 | 46.37 |

**Sample Availability:** Samples of the compounds are not available from the authors.

© 2018 by the authors. Submitted for possible open access publication under the terms and conditions of the Creative Commons Attribution (CC BY) license (http://creativecommons.org/licenses/by/4.0/).
